# Mathematical analysis of a two-strain tuberculosis model in Bangladesh

**DOI:** 10.1038/s41598-022-07536-2

**Published:** 2022-03-07

**Authors:** Md Abdul Kuddus, Emma S. McBryde, Adeshina I. Adekunle, Lisa J. White, Michael T. Meehan

**Affiliations:** 1 Australian Institute of Tropical Health and Medicine, James Cook University, Townsville, QLD Australia; 2 College of Medicine and Dentistry, James Cook University, Townsville, QLD Australia; 3 Big Data Institute, Li Ka Shing Centre for Health Information and Discovery, Nuffield Department of Medicine, University of Oxford, Oxford, UK; 4 Department of Mathematics, University of Rajshahi, Rajshahi, 6205 Bangladesh; 5 Decision Sciences Program, Victoria University, Melbourne, Australia

**Keywords:** Biological techniques, Computational biology and bioinformatics, Diseases, Mathematics and computing

## Abstract

Tuberculosis (TB) is an airborne infectious disease that causes millions of deaths worldwide each year (1.2 million people died in 2019). Alarmingly, several strains of the causative agent, *Mycobacterium tuberculosis* (MTB)—including drug-susceptible (DS) and drug-resistant (DR) variants—already circulate throughout most developing and developed countries, particularly in Bangladesh, with totally drug-resistant strains starting to emerge. In this study we develop a two-strain DS and DR TB transmission model and perform an analysis of the system properties and solutions. Both analytical and numerical results show that the prevalence of drug-resistant infection increases with an increasing drug use through amplification. Both analytic results and numerical simulations suggest that if the basic reproduction numbers of both DS (${\text{R}}_{{0{\text{s}}}}$) and DR (${\text{R}}_{{0{\text{r}}}}$) TB are less than one, i.e. $\max \left[ {{\text{R}}_{{0{\text{s}}}} ,{\text{ R}}_{{0{\text{r}}}} } \right] < 1,$ the disease-free equilibrium is asymptotically stable, meaning that the disease naturally dies out. Furthermore, if ${\text{R}}_{{0{\text{r}}}} > {\text{max}}\left[ {{\text{R}}_{{0{\text{s}}}} ,1} \right]$, then DS TB dies out but DR TB persists in the population, and if ${\text{R}}_{{0{\text{s}}}} > {\text{max}}\left[ {{\text{R}}_{{0{\text{r}}}} ,1} \right]$ both DS TB and DR TB persist in the population. Further, sensitivity analysis of the model parameters found that the transmission rate of both strains had the greatest influence on DS and DR TB prevalence. We also investigated the effect of treatment rates and amplification on both DS and DR TB prevalence; results indicate that inadequate or inappropriate treatment makes co-existence more likely and increases the relative abundance of DR TB infections.

## Introduction

Tuberculosis (TB) is a bacterial infectious disease that causes millions of deaths worldwide each year. In 2019, the World Health Organization (WHO) estimated there were approximately 10.0 million new cases of TB, and 1.2 million individuals died from TB disease^1^. Most of the observed cases in 2019 happened in Asia (44%) and Africa (24%), and 87% of tuberculosis deaths occurred in low- and middle-income countries^1^.

TB spreads from person to person through airborne particles that contain *Mycobacterium tuberculosis* (MTB)^2^. Healthy people become infected with TB through inhalation of the TB bacilli. Once infected, TB bacteria can live in the body without causing detectable clinical symptoms; this is called latent TB infection (LTBI)^3^. However, the latent MTB bacteria can activate, which may take weeks, months or the whole life of the infected individual^4^. Around 5–15% of the people infected with TB bacilli progress to TB disease in their lifetime^4^.

Currently, drug-resistant (DR) TB is emerging as the greatest threat to TB control globally^5^. Drug-resistant (DR) TB is well-defined as TB that is resistant to isoniazid and rifampicin (the two most efficient and commonly used first line-drugs), with or without additional resistance to supplementary first line-drugs^5^. The higher costs, and the longer and more toxic regimens associated with DR TB treatment place substantial stress on health systems^6^. Inadequate treatment of DR TB may create even more resistance to the drug used; this has been termed the amplification effect of short-course combination therapy^7^. Ongoing transmission of DR TB strains in a population also generates new DR TB cases^8^.

Mathematical modelling is an important tool to explore the dynamics of TB and can provide useful insights into the performance of various TB control strategies^9–12^. In the last few decades, numerous mathematicians, statisticians and biologists have established different transmission dynamic models of TB. For instance, Murphy et al. (2002) used a modified Susceptible Exposed Infected (SEI) model to examine the special effects of genetic susceptibility and demographic features on TB epidemiology in a heterogeneous population, comparing the prevalence and incidence in India and the United States of America (USA)^13^. Kim et al. (2014) developed a mathematical model for TB with exogenous reinfection, examining the present situation of active TB incidence in Korea^14^. Liu et al. (2010) developed a TB model with seasonality to describe TB incidence rates with periodic properties in a mainland city of China^15^. A 10-compartment TB model constructed by Trauer et al. (2014) modelled limited vaccine effectiveness, reinfection, DR TB, and de novo resistance through treatment^16^. This study showed that the model could not be calibrated to the projected incidence rate without allowing for reinfection, which was modelled as a reversion to early latency, which has a higher rate of progression to disease compared with late latency.

A theoretical framework for TB control strategies is developed by Blower et al*.* (1996) to assess the effect of chemoprophylaxis and treatment for eradication of TB epidemics. Results show that increasing chemoprophylaxis and treatment rates will decrease the severity of TB epidemics and reduce the probability that a latently infected individual will progress to active TB. This study recommended that in developing countries treatment failure rates must be lower than in developed countries in order to control TB outbreaks^17^. Kuddus et al*.* (2020) developed a two-strain TB model to determine the optimal time-varying combination of distancing, latent case finding, case holding and active case finding control strategies that minimize tuberculosis incidence over a fixed timespan. The major finding of this study was that combining two or more intervention strategies is more cost-effective compared to single-intervention strategies^12^. In 2010, Huo et al*.* developed a two-strain transmission dynamic model with susceptible, exposed and infected compartments that included drug-sensitive and drug-resistant TB^18^. Dynamical system analysis was used to ascertain the global stability of the disease-free equilibrium and a mono-existent equilibrium. Results show that the drug-sensitive and drug-resistant TB will die out if the both basic reproduction numbers are less than one, otherwise an epidemic occurs^18^.

TB is an ancient disease, which is still a major public health problem in Bangladesh. The problem is aggravated by the increasing drug-resistant mutations that arise through inadequate or poor quality treatment. To evaluate the threat of genetic variations of DS and DR TB strains, we present a two-strain (DS and DR TB) Susceptible-Latent-Infectious-Removed-Susceptible (SLIRS) TB model and examine the development and transmission of DR TB. We acknowledge the possibility that an individual’s position swaps from DS TB at primary appearance to DR TB at follow-up. This is the manner by which DR TB typically emerges in a population and is intended to replicate the phenotypic phenomenon of acquired drug resistance, known as amplification. The model can be used to examine the co-existent or competitive phenomena between DS and DR TB strains.

In this study, we perform a rigorous analytical and numerical analysis of our novel two-strain TB model properties and solutions from both the mathematical and biological perspectives. For an individual, we apply the next generation matrix technique (which is effectively a table describing the number of new infections generated by each individual infected with a particular strain) to determine analytic expressions for the basic reproduction numbers of the DS TB and DR TB strains. We find that these are key determinates for regulating model dynamics. We also present the mandatory conditions for the stability of the infection-free state, as well as infection mono-existence and co-existence.

To enhance and verify the analytical analysis, we apply numerical procedures to solve the system equations and investigate the dynamic epidemic trajectory for a variety of initial conditions and plausible parameter values. From the analytical and numerical viewpoints, the local and global stability of the infection-free equilibrium and mono-existent equilibrium are examined through standard dynamical systems analysis techniques^19^. The co-existent endemic equilibrium is also examined numerically. Following this, we implement a sensitivity analysis to examine the model parameters that have the greatest effect on DS, DR and total TB prevalence.

## Methods and materials

### Model formulation

We developed a deterministic mathematical model of the transmission of DS and DR TB strains between the following mutually exclusive compartments: susceptible ${\text{S}}\left( {\text{t}} \right)$, uninfected individuals who are susceptible to TB infection; those exposed to TB that become latently infected ${\text{L}}_{{\text{i}}} \left( {\text{t}} \right)$ (where the subscript ${\text{i}} = {\text{s}},{\text{r}}$ refers to quantities associated with drug-susceptible, ${\text{s}}$, and drug-resistant, ${\text{r}}$, TB infection), representing those who are infected and have not yet developed active TB; infectives ${\text{I}}_{{\text{i}}} \left( {\text{t}} \right)$, comprising individuals with active TB that are capable of transmitting the infection; the recovered ${\text{R}}\left( {\text{t}} \right)$, who were previously infected and successfully treated and assumed to be temporarily immune to reinfection. We assume that DR TB is initially generated through inadequate and poor treatment of DS TB and could subsequently be transmitted to other individuals. Individuals may also return to the susceptible compartment following recovery at the constant per-capita rate ${\upgamma }$ due to loss of immunity. The total population size ${\text{N}}\left( {\text{t}} \right)$, is given by

1 $${\text{N}}\left( {\text{t}} \right) = {\text{S}}\left( {\text{t}} \right) + {\text{L}}_{{\text{s}}} \left( {\text{t}} \right) + {\text{I}}_{{\text{s}}} \left( {\text{t}} \right) + {\text{L}}_{{\text{r}}} \left( {\text{t}} \right) + {\text{I}}_{{\text{r}}} \left( {\text{t}} \right) + {\text{R}}\left( {\text{t}} \right).$$

**Figure 1:**
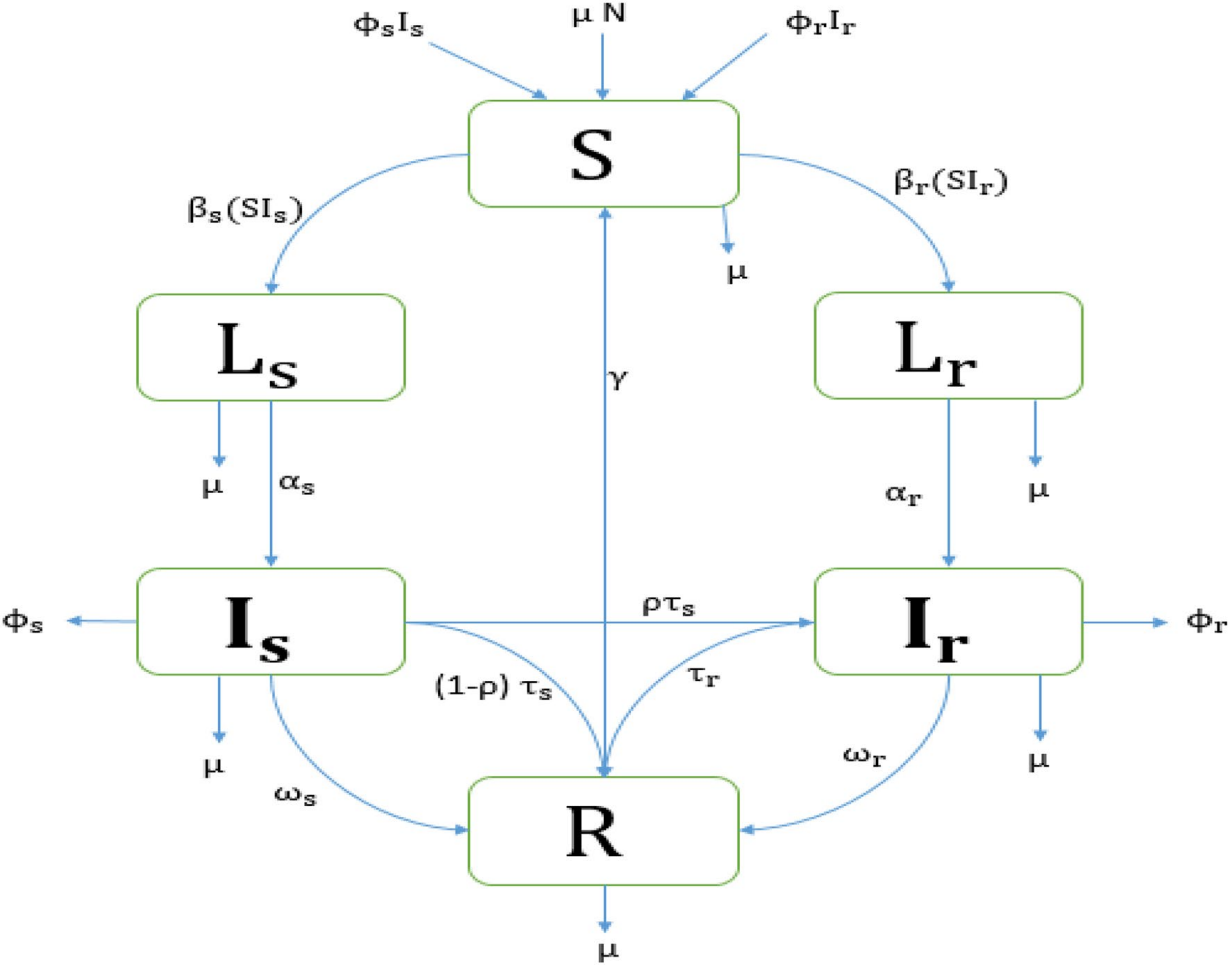
Flow chart of the TB compartmental mathematical model showing six states and the transitions in and out of each state in a closed population (no migration). Here, ${\text{N}} =$ Total population, ${\text{S}} =$ Susceptible population, ${\text{L}} =$ Latent population, ${\text{I}} =$ Infected population, ${\text{R}} =$ Recovered population, ${\upmu } =$ Birth rate / Death rate, ${\upbeta } =$ Contact rate/Transmission rate, ${\upalpha } =$ Progression rate, $\phi =$ Disease-related death rate, ${\uptau } =$ Treatment rate, ${\upomega } =$ Recovery rate, ${\uprho } =$ Proportion of amplification and ${\upgamma } =$ Rate of losing immunity. Subscripts ${\text{s}}$ and ${\text{r}}$ denote DS and DR quantities, respectively.

Individuals in the different compartments suffer from natural death at the same constant rate ${\upmu }$ and active TB cases in ${\text{I}}_{{\text{i}}} \left( {{\text{i}} = {\text{s}},{\text{r}}} \right)$ experience disease-related death at a rate $\upphi _{{\text{i}}} \left( {{\text{i}} = {\text{s}},{\text{r}}} \right)$. To ensure the population size remains constant, we replace all deaths as newborns in the susceptible compartment. Individuals in the ${\text{S}}$ compartment may be infected with a circulating MTB strain ${\text{i }}\left( {{\text{i}} = {\text{s}},{\text{r}}} \right)$ at a time dependent rate ${\uplambda }_{{\text{i}}} \left( {\text{t}} \right) = {\upbeta }_{{\text{i}}} {\text{I}}_{{\text{i}}} \left( {\text{t}} \right)$ where ${\upbeta }_{{\text{i}}}$ is the transmission rate between infected and susceptible individuals. Once infected with strain ${\text{I}}_{{\text{i}}} \left( {{\text{i}} = {\text{s}},{\text{r}}} \right)$, individuals move to the latently infected compartment ${\text{L}}_{{\text{i}}}$. A proportion of those with latent infections progress to active TB as a result of endogenous reactivation of the latent bacilli at rate ${\upalpha }_{{\text{i}}}$. Individuals with drug sensitive and DR active TB, ${\text{I}}_{{\text{i}}}$, may eventually be detected and treated at rates ${\uptau }_{{\text{s}}}$ and ${\uptau }_{{\text{r}}}$ for DS and DR TB, respectively. A proportion $\left( {1 - {\uprho }} \right)$ of the treated DS active TB recover to move into the recovered compartment ${\text{R}}$, and the complementary proportion ${\uprho }$ develop drug resistance due to incomplete treatment or lack of strict compliance in the use of first-line drugs (drugs used to treat the DS forms of TB) to move into compartment ${\text{I}}_{{\text{r}}}$. Furthermore, individuals recover naturally at a rate ${\upomega }_{{\text{i}}}$, moving from ${\text{I}}_{{\text{i}}}$ to ${\text{R}}$. The model flow diagram is presented in Fig. 1. The parameters and their values are given in Table 1.

**Table 1:** Depiction and estimation of parameters.

Parameters	Description	Estimated value	
${\text{N}}$	Population in 2015	159,000,000	^29^
${\upmu }$	Birth/death rate	$\frac{1}{70}{ }$ per year	^30^
${\upbeta }_{{\text{s}}}$	Transmission rate for DS TB	$1.57 \times 10^{ - 8}$	Fitted
${\upbeta }_{{\text{r}}}$	Transmission rate for DR TB	$6.25 \times 10^{ - 9}$	Fitted
${\upalpha }_{{\text{s}}}$	Progression rate from ${\text{L}}_{{\text{s}}}$ to ${\text{I}}_{{\text{s}}}$	0.129 per year	^16^
${\upalpha }_{{\text{r}}}$	Progression rate from ${\text{L}}_{{\text{r}}}$ to ${\text{I}}_{{\text{r}}}$	0.129 per year	^16^
${\upomega }_{{\text{s}}}$	Recovery rate for DS TB	0.287 per year	^12^
${\upomega }_{{\text{r}}}$	Recovery rate for DR TB	0.12 per year	^12^
${\uprho }$	Proportion of treated patients who amplify	0.07 per year	^11^
$\phi_{{\text{s}}}$	Disease related death rate for DS TB	0.37 over 3 years	^16^
$\phi_{{\text{r}}}$	Disease related death rate for DR TB	0.37 over 3 years	^16^
${\uptau }_{{\text{s}}}$	Treatment rate for DS TB	0.94 per year	^20^
${\uptau }_{{\text{r}}}$	Treatment rate for DR TB	0.78 per year	^20^
${\upgamma }$	Rate of losing immunity	0.10 per year	^12^

The transmission of DS and DR TB is given by the following deterministic system of nonlinear ordinary differential equations that describe the model:

2 $$\frac{{{\text{dS}}}}{{{\text{dt}}}} = {\mu N} - {\upbeta }_{{\text{s}}} {\text{I}}_{{\text{s}}} {\text{S}} - {\upbeta }_{{\text{r}}} {\text{I}}_{{\text{r}}} {\text{S}} - {\mu S} + {\gamma R} + \phi_{{\text{s}}} {\text{I}}_{{\text{s}}} + \phi_{{\text{r}}} {\text{I}}_{{\text{r}}} ,$$

3 $$\frac{{{\text{dL}}_{{\text{s}}} }}{{{\text{dt}}}} = {\upbeta }_{{\text{s}}} {\text{I}}_{{\text{s}}} {\text{S}} - {\upalpha }_{{\text{s}}} {\text{L}}_{{\text{s}}} - {\mu L}_{{\text{s}}} ,$$

4 $$\frac{{{\text{dI}}_{{\text{s}}} }}{{{\text{dt}}}} = {\upalpha }_{{\text{s}}} {\text{L}}_{{\text{s}}} - {\upomega }_{{\text{s}}} {\text{I}}_{{\text{s}}} - {\mu I}_{{\text{s}}} - {\uptau }_{{\text{s}}} {\text{I}}_{{\text{s}}} - \phi_{{\text{s}}} {\text{I}}_{{\text{s}}} ,$$

5 $$\frac{{{\text{dL}}_{{\text{r}}} }}{{{\text{dt}}}} = {\upbeta }_{{\text{r}}} {\text{I}}_{{\text{r}}} {\text{S}} - {\upalpha }_{{\text{r}}} {\text{L}}_{{\text{r}}} - {\mu L}_{{\text{r}}} ,$$

6 $$\frac{{{\text{dI}}_{{\text{r}}} }}{{{\text{dt}}}} = {\upalpha }_{{\text{r}}} {\text{L}}_{{\text{r}}} - {\upomega }_{{\text{r}}} {\text{I}}_{{\text{r}}} - {\mu I}_{{\text{r}}} + {{\rho \tau }}_{{\text{s}}} {\text{I}}_{{\text{s}}} - \phi_{{\text{r}}} {\text{I}}_{{\text{r}}} - {\uptau }_{{\text{r}}} {\text{I}}_{{\text{r}}} ,$$

7 $$\frac{{{\text{dR}}}}{{{\text{dt}}}} = {\upomega }_{{\text{s}}} {\text{I}}_{{\text{s}}} + {\upomega }_{{\text{r}}} {\text{I}}_{{\text{r}}} - {\gamma R} - {\mu R} + \left( {1 - {\uprho }} \right){\uptau }_{{\text{s}}} {\text{I}}_{{\text{s}}} + {\uptau }_{{\text{r}}} {\text{I}}_{{\text{r}}} .$$

with non-negative initial conditions for the system of differential equations above, it is easy to express that each of the state variables remain non-negative for all ${\text{t}} > 0$. Moreover, from Eqs. (2)–(7), we find that the total population size, ${\text{N}}\left( {\text{t}} \right)$ satisfies

$${\text{N}}\left( {\text{t}} \right) = {\text{constant}}{.}$$

Given the constant population size and positivity of solutions it generally follows that each of the compartment states ${\text{S}},{\text{ L}},{\text{ I}},{ }$ etc. are bounded. Therefore, based on the considerations, the feasible region for the system Eqs. (2)–(7) is given by

c $${\text{D}} = \left\{ {{ }\left( {{\text{S}},{\text{ L}}_{{\text{s}}} ,{\text{ I}}_{{\text{s}}} ,{\text{ L}}_{{\text{r}}} ,{\text{ I}}_{{\text{r}}} ,{\text{R}}} \right) \in {\mathbb{R}}_{ + }^{6} { }:{\text{S}} + {\text{L}}_{{\text{s}}} + {\text{I}}_{{\text{s}}} + {\text{L}}_{{\text{r}}} + {\text{I}}_{{\text{r}}} + {\text{R}} = {\text{N}}} \right\}.$$

### Estimation of model parameters

**Figure 2:**
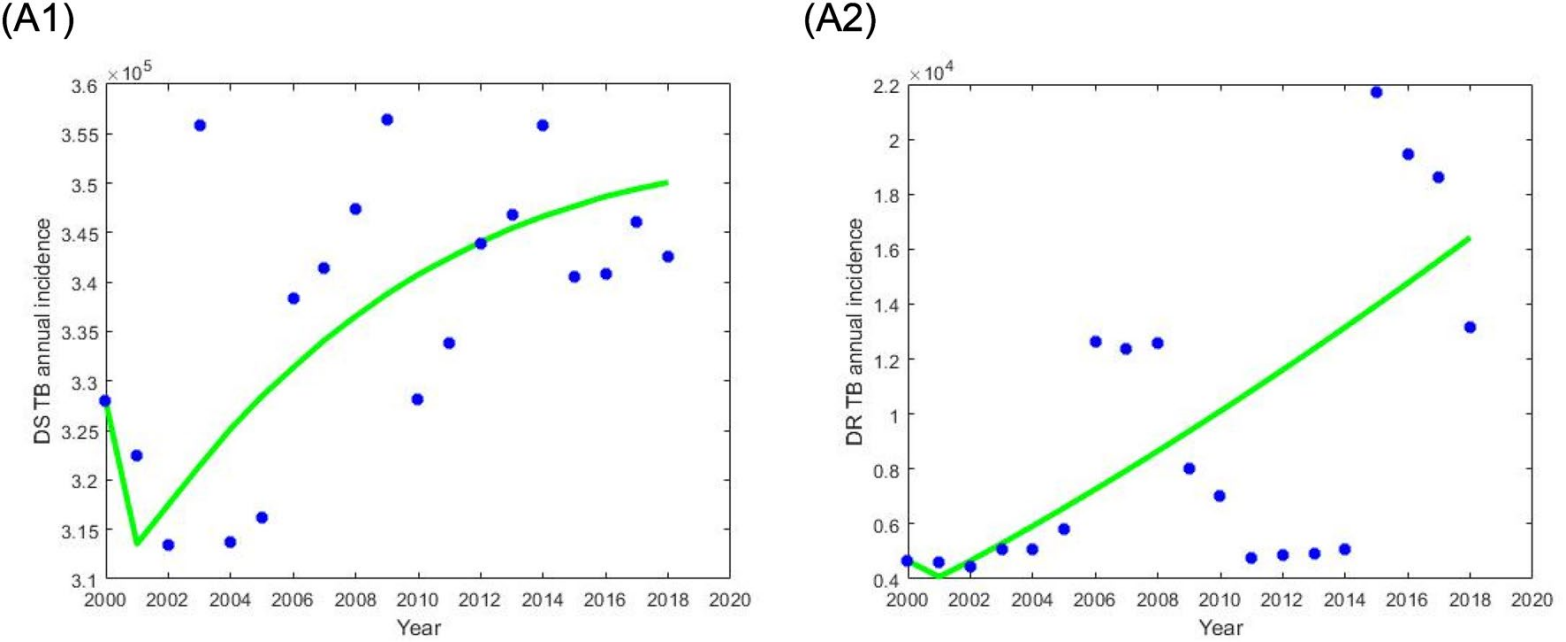
WHO reported DS and DR TB annual incidence data (blue dot) and the corresponding best fit (green solid curve) of our proposed model: (left) DS TB and (right) DR TB.

Most parameters were estimated from the global literature or taken from National TB Control Program (NTP) evaluation (Table 1). We determined the total population of Bangladesh in 2015 is about 159,000,000 based on the National TB Control Program (NTP) reports. The natural death rate $\left( {\upmu } \right)$ was considered as the inverse of life expectancy (70 years) of Bangladesh. According to the data from the WHO report^20^ the treatment success rates for DS $\left( {{\uptau }_{{\text{s}}} } \right)$ and DR $\left( {{\uptau }_{{\text{m}}} } \right)$ TB in Bangladesh were about 94% and 78% respectively. We estimated two critical residual model parameters, ${\upbeta }_{{\text{s}}}$ and ${\upbeta }_{{\text{m}}}$ by fitting our model to the DS and DR TB incidence data in Bangladesh taken from the WHO and NTP report from 2000 to 2018^1,5,21^. The best fitted (see Fig. 2) parameter values ${\upbeta }_{{\text{s}}}$ and ${\upbeta }_{{\text{m}}}$ were obtained, using the least-square optimization method to minimize the squared error between the modelled and actual number of DS and DR TB annual incident cases. Details are given in the supplementary materials.

### Sensitivity analysis

We calculated the Partial Rank Correlation Coefficients (PRCCs) between each of the model parameters and several outcome measures of significance, a global sensitivity analysis method using Latin Hypercube Sampling (LHS). Explicitly, a uniform distribution is allocated from half to fourfold the baseline value (see Table 1) for each model parameter (i.e.$\upbeta _{{\text{s}}} , \upalpha _{{\text{s}}} ,\upomega _{{\text{s}}} , \upphi _{{\text{s}}} ,\uptau _{{\text{s}}} , \upbeta _{{\text{r}}} ,\;\upalpha _{{\text{r}}} ,\upomega _{{\text{r}}} , \upphi _{{\text{r}}} , \uptau _{{\text{r}}} , \uprho$ and $\upgamma$) and a total of 10,000,000 random draws are taken for each. The model is then simulated for each of the 10,000,000 parameter sets and relevant outputs such as disease prevalence and incidence are recorded. Here the model outputs we considered were DS TB $\left( {{\text{I}}_{{\text{s}}} } \right)$, DR TB $\left( {{\text{I}}_{{\text{r}}} } \right)$ and total TB $\left( {{\text{I}}_{{\text{s}}} + {\text{I}}_{{\text{r}}} } \right)$ prevalence at equilibrium.

From the analytical formula for ${\text{R}}_{{0{\text{s}}}}$ and ${\text{R}}_{{0{\text{r}}}}$, the sensitivity indices (measuring the relative change in a variable when a parameter changes) ${\Upsilon }_{{\text{j}}}^{{\text{i}}}$ can be derived applying the technique in^22^ to each of the model parameters. For example, for ${\upbeta }_{{\text{s}}}$ we have:

$${\Upsilon }_{{{\upbeta }_{{\text{s}}} }}^{{{\text{R}}_{{0{\text{s}}}} }} = { }\frac{{\partial {\text{R}}_{{0{\text{s}}}} }}{{\partial {\upbeta }_{{\text{s}}} }} \times { }\frac{{{\upbeta }_{{\text{s}}} }}{{{\text{R}}_{{0{\text{s}}}} }}.$$

Here ${\Upsilon }_{{{\upbeta }_{{\text{s}}} }}^{{{\text{R}}_{{0{\text{s}}}} }}$ is the sensitivity index for the basic reproduction number ${\text{R}}_{{0{\text{s}}}}$, as we vary ${\upbeta }_{{\text{s}}}$.

### Ethical approval

The study was based on aggregated DS and DR TB surveillance data taken from the World Health Organization and the National TB Control Program in Bangladesh^1,5,21^. No confidential information was involved because analyses were performed at the aggregate level. All of the methods were conducted in accordance with the approved research protocol. The research protocol was approved by the James Cook University human ethics approval board, H7300.

## Results

### Basic reproduction number

The model has four infected states: ${\text{L}}_{{\text{s}}} ,{\text{I}}_{{\text{s}}} ,{\text{L}}_{{\text{r}}} ,{\text{I}}_{{\text{r}}}$, and two uninfected states: S and R. At the infection-free steady state, ${\text{L}}_{{\text{s}}}^{*} = {\text{I}}_{{\text{s}}}^{*} = {\text{L}}_{{\text{r}}}^{*} = {\text{I}}_{{\text{r}}}^{*} = {\text{R}}^{*} = 0,$ hence ${\text{S}}^{*} = {\text{N}}$. To calculate the basic reproduction numbers of the DS and DR TB strains we follow^23^ and focus on the linearized infection subsystem derived from Eqs. (2)-(7):

8 $$\frac{{{\text{dL}}_{{\text{s}}} }}{{{\text{dt}}}} = {\upbeta }_{{\text{s}}} {\text{I}}_{{\text{s}}} {\text{N}} - {\upalpha }_{{\text{s}}} {\text{L}}_{{\text{s}}} - {\mu L}_{{\text{s}}} ,$$

9 $$\frac{{{\text{dI}}_{{\text{s}}} }}{{{\text{dt}}}} = {\upalpha }_{{\text{s}}} {\text{L}}_{{\text{s}}} - {\upchi }_{{\text{s}}} {\text{I}}_{{\text{s}}} ,$$

10 $$\frac{{{\text{dL}}_{{\text{r}}} }}{{{\text{dt}}}} = {\upbeta }_{{\text{r}}} {\text{I}}_{{\text{r}}} {\text{N}} - {\upalpha }_{{\text{r}}} {\text{L}}_{{\text{r}}} - {\mu L}_{{\text{r}}} ,$$

11 $$\frac{{{\text{dI}}_{{\text{r}}} }}{{{\text{dt}}}} = {\upalpha }_{{\text{r}}} {\text{L}}_{{\text{r}}} - {\upchi }_{{\text{r}}} {\text{I}}_{{\text{r}}} + {{\rho \tau }}_{{\text{s}}} {\text{I}}_{{\text{s}}} ,$$

where ${\upchi }_{{\text{s}}} = {\upomega }_{{\text{s}}} + \phi_{{\text{s}}} + {\uptau }_{{\text{s}}} + {\mu }$ and ${\upchi }_{{\text{m}}} = {\upomega }_{{\text{r}}} + \phi_{{\text{r}}} + {\uptau }_{{\text{r}}} + {\upmu }$ are the total removal rates from the DS and DR active TB infection states respectively.

Here, the ODEs (8)–(11) represent the changes in the infected states about the infection-free equilibrium (that is, when assuming the reduction in the susceptible population as a result of infection is negligible).

By setting ${\mathbf{X}}^{{\text{T}}} = \left( {{\text{L}}_{{\text{s}}} ,{\text{I}}_{{\text{s}}} ,{\text{L}}_{{\text{r}}} ,{\text{I}}_{{\text{r}}} } \right)^{{\text{T}}}$, where T denotes transpose, now we can write the infection subsystem in the following form

12 $${\dot{\mathbf{X}}} = \left( {T + {\Sigma }} \right){\mathbf{X}}.$$

The matrix $T$ corresponds to transmissions and the matrix ${\Sigma }$ to transitions. They are acquired from system $\left( 8 \right) - \left( {11} \right)$ by splitting the transmission events from other events. If we indicate to the infected states with indices ${\text{i}}$ and j, with ${\text{i}},{\text{j}} \in 1,2,3,4,{ }$ then entry $T_{ij}$ is the rate per unit time at which persons in infected state ${\text{j}}$ give rise to persons in infected state ${\text{i}}$ in the system. Hence, for the subsystem $\left( 8 \right) - \left( {11} \right)$ we obtain

$$T = \left( {\begin{array}{*{20}c} {\begin{array}{*{20}c} {{ }0} & {{\upbeta }_{{\text{s}}} {\text{N}}} \\ \end{array} } & 0 & 0 \\ {\begin{array}{*{20}c} 0 & 0 \\ \end{array} } & 0 & 0 \\ {\begin{array}{*{20}c} {\begin{array}{*{20}c} 0 \\ 0 \\ \end{array} } & {\begin{array}{*{20}c} 0 \\ 0 \\ \end{array} } \\ \end{array} } & {\begin{array}{*{20}c} 0 \\ 0 \\ \end{array} } & {\begin{array}{*{20}c} {{\upbeta }_{{\text{r}}} {\text{N}}} \\ 0 \\ \end{array} } \\ \end{array} { }} \right)\;{\text{and}}\;{\Sigma } = \left( {\begin{array}{*{20}c} { - \left( {{\upalpha }_{{\text{s}}} + {\upmu }} \right)} & 0 & 0 \\ {{\upalpha }_{{\text{s}}} } & { - {\upchi }_{{\text{s}}} } & 0 \\ {\begin{array}{*{20}c} 0 \\ 0 \\ \end{array} } & {\begin{array}{*{20}c} 0 \\ {{{\rho \tau }}_{{\text{s}}} } \\ \end{array} } & {\begin{array}{*{20}c} { - \left( {{\upalpha }_{{\text{r}}} + {\upmu }} \right)} \\ {{\upalpha }_{{\text{r}}} } \\ \end{array} } \\ \end{array} { }\begin{array}{*{20}c} 0 \\ 0 \\ {\begin{array}{*{20}c} 0 \\ { - {\upchi }_{{\text{r}}} } \\ \end{array} } \\ \end{array} } \right)$$

The next generation matrix, ${\text{K}}$, is given by^24^ (note the essential minus sign)

$${\text{K}} = - T\Sigma ^{{ - 1}} = T\left( { - \Sigma ^{{ - 1}} } \right) = \left( {\begin{array}{*{20}c} {\frac{{{\text{N}}{\upalpha }_{{\text{s}}} {\upbeta }_{{\text{s}}} }}{{\left( {{\upalpha }_{{\text{s}}} + {\upmu }} \right){\upchi }_{{\text{s}}} }}} & {\frac{{{\text{N}}{\upbeta }_{{\text{s}}} }}{{{\upchi }_{{\text{s}}} }}} & {\begin{array}{*{20}c} {0~~~~~~~~~~~~~~~~~~} & 0 \\ \end{array} } \\ 0 & 0 & {\begin{array}{*{20}c} {0~~~~~~~~~~~~~~~~~~} & 0 \\ \end{array} } \\ {\begin{array}{*{20}c} {\frac{{{\text{N}}{\upalpha }_{{\text{s}}} {\upbeta }_{{\text{r}}} {\uprho \uptau }_{{\text{s}}} }}{{\left( {{\upalpha }_{{\text{s}}} + {\upmu }} \right){\upchi }_{{\text{s}}} {\upchi }_{{\text{r}}} }}} \\ 0 \\ \end{array} } & {\begin{array}{*{20}c} {\frac{{{\text{N}}{\upbeta }_{{\text{r}}} {\uprho \uptau }_{{\text{s}}} }}{{{\upchi }_{{\text{s}}} {\upchi }_{{\text{r}}} }}} \\ 0 \\ \end{array} } & {\begin{array}{*{20}c} {\begin{array}{*{20}c} {\frac{{{\text{N}}{\upalpha }_{{\text{r}}} {\upbeta }_{{\text{r}}} }}{{\left( {{\upalpha }_{{\text{r}}} + {\upmu }} \right){\upchi }_{{\text{r}}} }}} & {\frac{{{\text{N}}{\upbeta }_{{\text{r}}} }}{{{\upchi }_{{\text{r}}} }}} \\ \end{array} } \\ {\begin{array}{*{20}c} {0~~~~~~~~~~~~~~~~~} & 0 \\ \end{array} } \\ \end{array} } \\ \end{array} ~~} \right).$$

The leading eigenvalues of the next generation matrix $\left( {\text{K}} \right)$ are the basic reproduction numbers for DS and DR TB; they describe the average number of secondary infections generated by a single infected individual. Therefore the basic reproduction number for DS and DR TB are:

a $${\text{R}}_{{0{\text{s}}}} = \frac{{{\text{N}}{\upalpha }_{{\text{s}}} {\upbeta }_{{\text{s}}} }}{{\left( {{\upalpha }_{{\text{s}}} + {\upmu }} \right){\upchi }_{{\text{s}}} }},$$

and

b $${\text{R}}_{{0{\text{r}}}} = \frac{{{\text{N}}{\upalpha }_{{\text{r}}} {\upbeta }_{{\text{r}}} }}{{\left( {{\upalpha }_{{\text{r}}} + {\upmu }} \right){\upchi }_{{\text{r}}} }}.$$

Here $\frac{{{\upalpha }_{{\text{s}}} }}{{\left( {{\upalpha }_{{\text{s}}} + {\upmu }} \right)}}$ and $\frac{{{\upalpha }_{{\text{r}}} }}{{\left( {{\upalpha }_{{\text{r}}} + {\upmu }} \right)}}$ are the probability of transitioning from the latent compartment to the infectious compartment of the DS and DR strains respectively. Further, $\frac{1}{{{\upchi }_{{\text{s}}} }}$ and $\frac{1}{{{\upchi }_{{\text{r}}} }}$ represent the time spent by infectious individuals in states ${\text{I}}_{{\text{s }}}$ and ${\text{I}}_{{\text{r }}}$ respectively.

The quantities ${\text{R}}_{{0{\text{s}}}}$ and ${\text{R}}_{{0{\text{m}}}}$ represents the expected number of secondary cases for DS and DR TB produced by the single infectious introduced into a completely susceptible population. Remarkably we identify that the basic reproduction numbers of DS TB $({\text{R}}_{{0{\text{s}}}} )$ and DR TB $\left( {{\text{R}}_{{0{\text{r}}}} } \right)$ are completely independent of the proportion of amplification ${\uprho }$ ^24^.

We provide detailed analysis of the proposed TB model (2)–(7) for the existence and stability of disease-free $\left( {{\text{E}}^{*} } \right)$, mono-existent $({\text{E}}^{ \wedge } )$ and co-existent $\left( {{\text{E}}^{\dag } } \right)$ endemic equilibrium points in the supplementary materials: existence of equilibria section and stability analysis section.

In summary the disease-free equilibrium is globally asymptotically stable when ${\text{max}}\left[ {{\text{R}}_{{0{\text{s}}}} ,{\text{R}}_{{0{\text{r}}}} } \right] < 1$. We demonstrate that the mono-existent equilibrium is locally stable provided ${\text{R}}_{{0{\text{r}}}} > {\text{max}}\left[ {1,{\text{ R}}_{{0{\text{s}}}} } \right]$ (see supplementary materials: stability analysis section).

**Figure 3:**
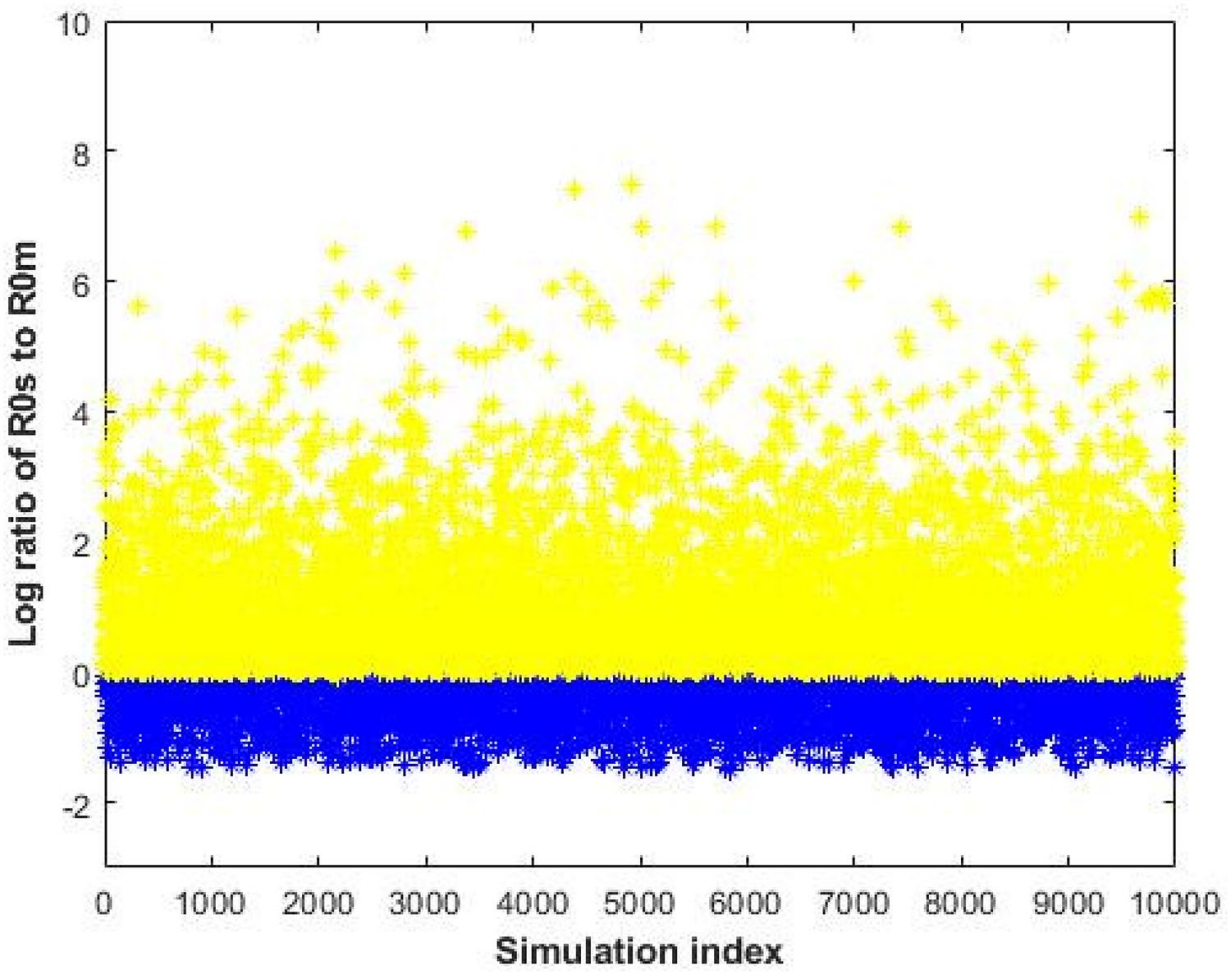
Stability of the co-existent endemic equilibrium as a function of ${\text{R}}_{{0{\text{s}}}} /{\text{R}}_{{0{\text{m}}}}$ (as determined by the eigenvalues of the system Jacobian matrix). Random parameter draws leading to eigenvalues with exclusively negative real parts (i.e. stable) are colored yellow, whilst those leading to eigenvalues with at least one positive real part (i.e. unstable) are colored blue. All stable points (yellow) lie above the line ${\text{R}}_{{0{\text{s}}}} = {\text{R}}_{{0{\text{r}}}}$, and all unstable points (blue) lie below.

To establish the nature of the co-existence equilibrium, we implemented numerical analysis using the Monte Carlo method in^25^ to verify the conditions ${\text{R}}_{{0{\text{s}}}} > {\text{max}}\left[ {{\text{R}}_{{0{\text{r}}}} ,{ }1} \right]$ by calculating the real part of the eigenvalues of the Jacobian matrix evaluated at the co-existent endemic equilibrium $\left( {{\text{E}}^{\dag } } \right)$. According to ${\text{E}}^{\dag }$ the coordinates of the steady states can be expressed in terms of twelve of the model’s parameters $\left( {\upalpha _{{\text{s}}} , \upalpha _{{\text{r}}} , \upbeta _{{\text{s}}} , \upbeta _{{\text{r}}} , \upomega _{{\text{s}}} , \upomega _{{\text{r}}} , \upphi _{{\text{s}}} , \upphi _{{\text{r}}} , \uptau _{{\text{s}}} , \uptau _{{\text{r}}} , \uprho , \upgamma } \right)$ whose baseline values are given in Table 1. The sampling pool ${\mathbb{Q}} = \mathop \prod \limits_{{{\text{i}} = 1}}^{12} {\text{M}}_{{\text{i}}} \in {\mathbb{R}}_{ + }^{12}$ was defined by a Cartesian product of twelve closed intervals of the form ${\text{M}}_{{\text{i}}} = \left[ {{\text{m}}_{{\text{i}}} - {\theta m}_{{\text{i}}} ,{\text{ m}}_{{\text{i}}} + {\theta m}_{{\text{i}}} } \right]$ where each ${\text{m}}_{{\text{i}}} ,{\text{ i}} = 1, \ldots ,12$ stands for the baseline value of one parameter (see Table 1) and ${\uptheta } > 0$ defines the variation range. Our sampling comprised 10,000 confounding scenarios ${\text{Q}} = \left( {{\text{q}}_{1} ,{ } \ldots ,{\text{ q}}_{12} } \right) \in {\mathbb{Q}}$ where each ${\text{q}}_{{\text{i}}} \in {\text{M}}_{{\text{i}}} ,{\text{ i}} = 1,{ } \ldots ,12$ was randomly chosen for ${\uptheta } = 0.20$ (i.e. 20% deviation from the baseline values) under a uniform distribution with no correlation between parameters. The simulation results are presented in Fig. 3. Figure 3 showed that the co-existent endemic equilibrium is stable (yellow-coloured points) when ${\text{R}}_{{0{\text{s}}}} > {\text{max}}\left[ {{\text{R}}_{{0{\text{r}}}} ,{ }1} \right]$, otherwise unstable (blue-coloured points).

**Figure 4:**
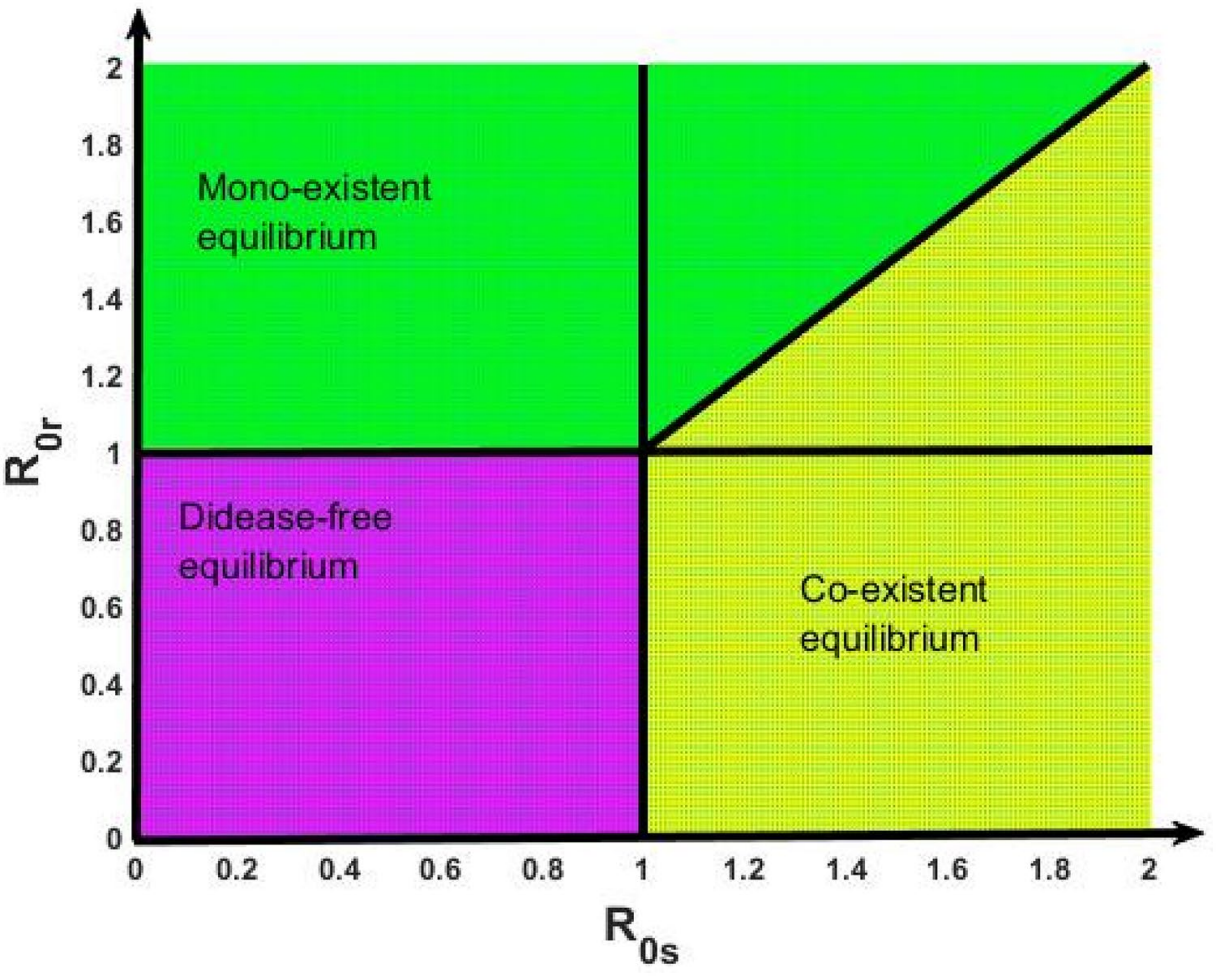
Graph shows the existence and local stability regions for the disease-free (magenta shaded region), mono-existent (green shaded region) and co-existent (yellow shaded region) equilibrium points.

Figure 4 represents the existence and local stability regions for the disease-free $\left( {{\text{E}}^{*} } \right)$, mono-existent (E^^^) and co-existent $\left( {{\text{E}}^{\dag } } \right)$ endemic equilibrium points corresponding to the drug-susceptible and drug-resistant TB basic reproduction numbers ${\text{R}}_{{0{\text{s}}}}$ and ${\text{R}}_{{0{\text{r}}}}$. The magenta shaded region indicates the disease-free equilibrium which is bounded by ${\text{max}}\left[ {{\text{R}}_{{0{\text{s}}}} ,{\text{R}}_{{0{\text{r}}}} } \right] < 1$. The green shaded area illustrates the mono-existent equilibrium where ${\text{R}}_{{0{\text{r}}}} > {\text{max}}\left[ {1,{\text{ R}}_{{0{\text{s}}}} } \right]$. Finally, the co-existent equilibrium in the yellow shaded region where ${\text{R}}_{{0{\text{s}}}} > {\text{max}}\left[ {{\text{R}}_{{0{\text{r}}}} ,{ }1} \right]$.

Various numerical simulations were carried out using the Matlab programming language to support the analytic results and to observe the impact of amplification and the DS TB treatment rate on total and DR TB prevalence. We applied different initial conditions for both DS and DR TB of all populations and obtained the stability results for the model equilibria, finding TB disease will ultimately die out from the population when the condition ${\text{ max}}\left[ {{\text{R}}_{{0{\text{s}}}} ,{\text{R}}_{{0{\text{r}}}} } \right] < 1,$ holds. The condition ${\text{R}}_{{0{\text{r}}}} > {\text{max}}\left[ {{\text{R}}_{{0{\text{s}}}} ,1} \right]$ implies that DS TB dies out but DR TB persists in the population. Furthermore, the condition ${\text{R}}_{{0{\text{s}}}} > {\text{max}}\left[ {{\text{R}}_{{0{\text{r}}}} ,1} \right]$ suggests that both DS TB and DR TB persist in the population (see supplementary materials: stability analysis section, Figures S1, S2 and S3).

Simulating the impact of public health effects through changes in model parameters. Public health interventions can be modelled by changing key parameters. For example, reducing contact between people can be modelled by changing ${\upbeta }_{{\text{s}}} { }\;{\text{or }}\;{\upbeta }_{{\text{r}}}$, detecting and treating infectious people can be modelled by increasing ${\uptau }_{{\text{s}}} \;{\text{or }}\;{\uptau }_{{\text{r}}}$. The resulting reproduction number after a change in a public health intervention or after a substantial proportion of the population is removed from the susceptible pool is often called the effective reproduction number.

**Figure 5:**
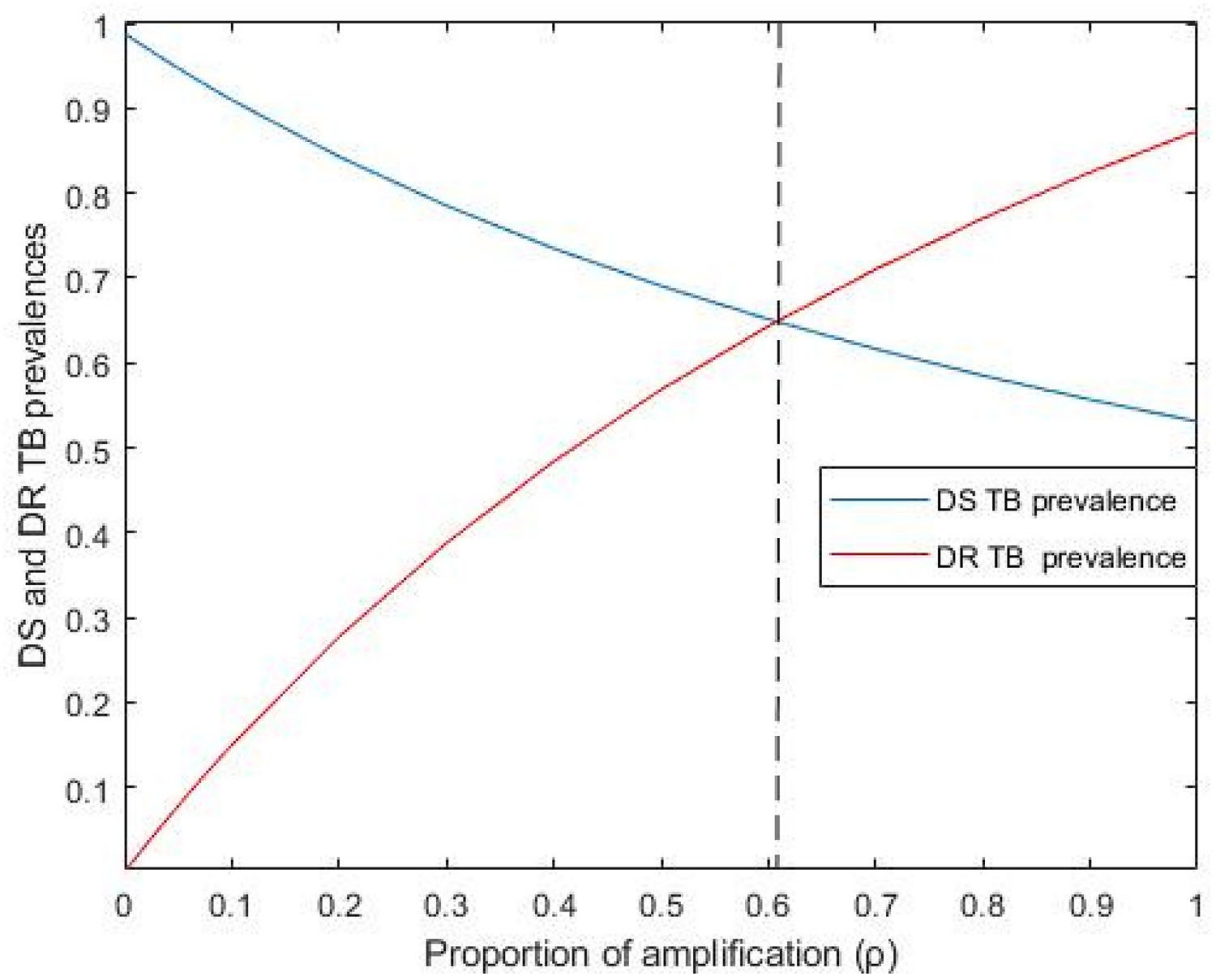
The impact of amplification $\left( {\uprho } \right)$ on DS and DR TB prevalence. All remaining parameters consider their baseline values as reported in Table 1.

Figure 5 shows the impact of amplification $$\left( {\uprho } \right)$$ on DS and DR TB prevalence in the first region ($$\uprho \underset{\raise0.3em\hbox{$\smash{\scriptscriptstyle\thicksim}$}}{ < } 0.6$$), DS TB initially dominates, but for $$\uprho \underset{\raise0.3em\hbox{$\smash{\scriptscriptstyle\thicksim}$}}{ > } 0.6$$ the DR TB has higher prevalence due to the amplification pathway when $${\text{R}}_{{0{\text{s}}}} > {\text{max}}\left[ {{\text{R}}_{{0{\text{r}}}} ,1} \right]$$. These outcomes implicate the use of drug treatment in the context of DR TB and note that treatment of DS TB can provoke the emergence of DR TB, even if it has lower reproductive fitness than the susceptible strain, including when its reproduction number is less than one. Further, under these situations, our investigation reveals that this emergence of DR TB will be overcome if the treatment rate is adequate to eliminate DS TB from the population (i.e. is able to achieve an effective reproduction number of DS TB less than one).

**Figure 6:**
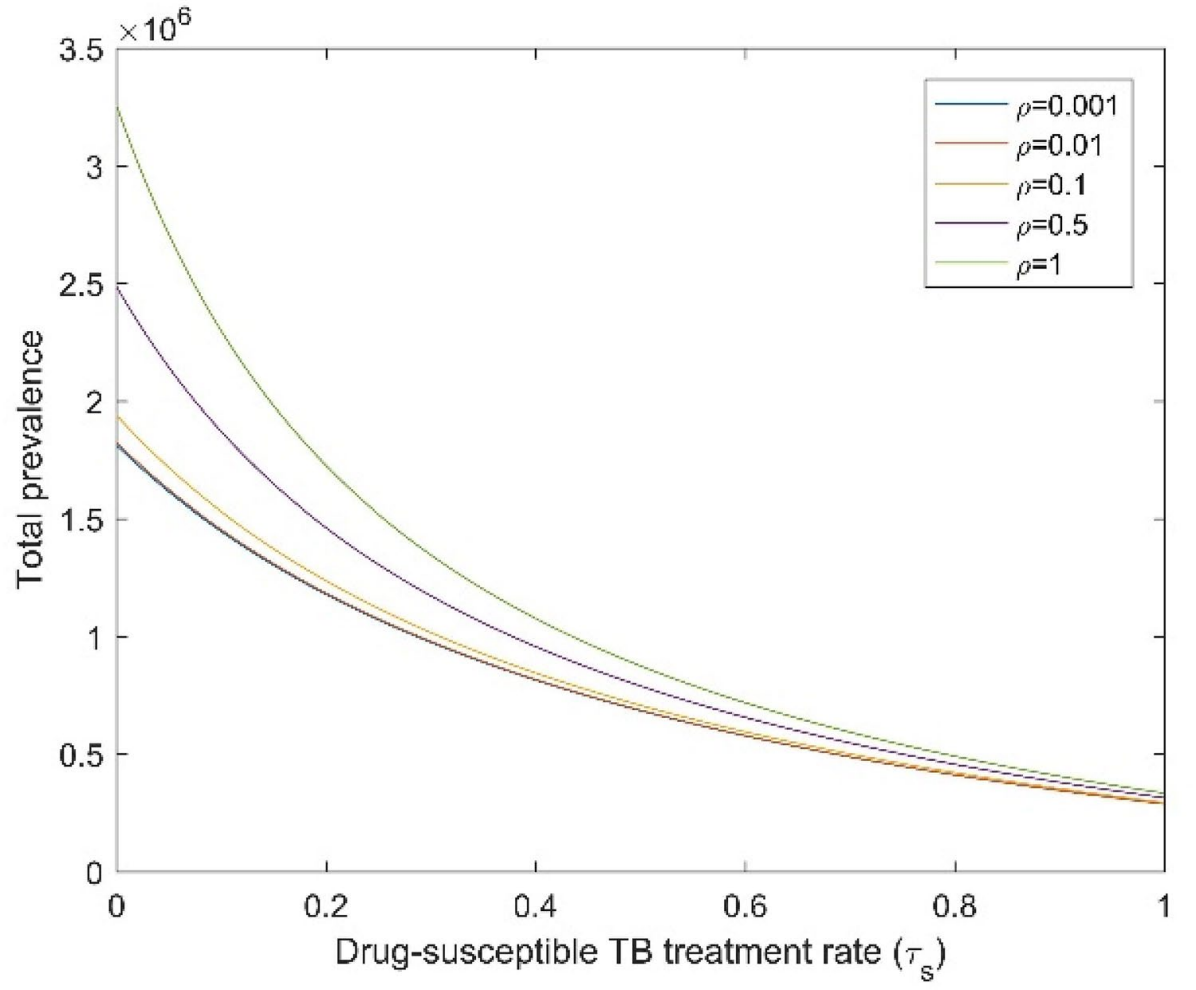
Effect of DS TB treatment rate (${\uptau }_{{\text{s}}}$) on total TB prevalence when ${\upbeta }_{{\text{s}}}$ and ${\upbeta }_{{\text{r}}}$ are fixed. All remaining parameters consider their baseline values as reported in Table 1.

**Figure 7:**
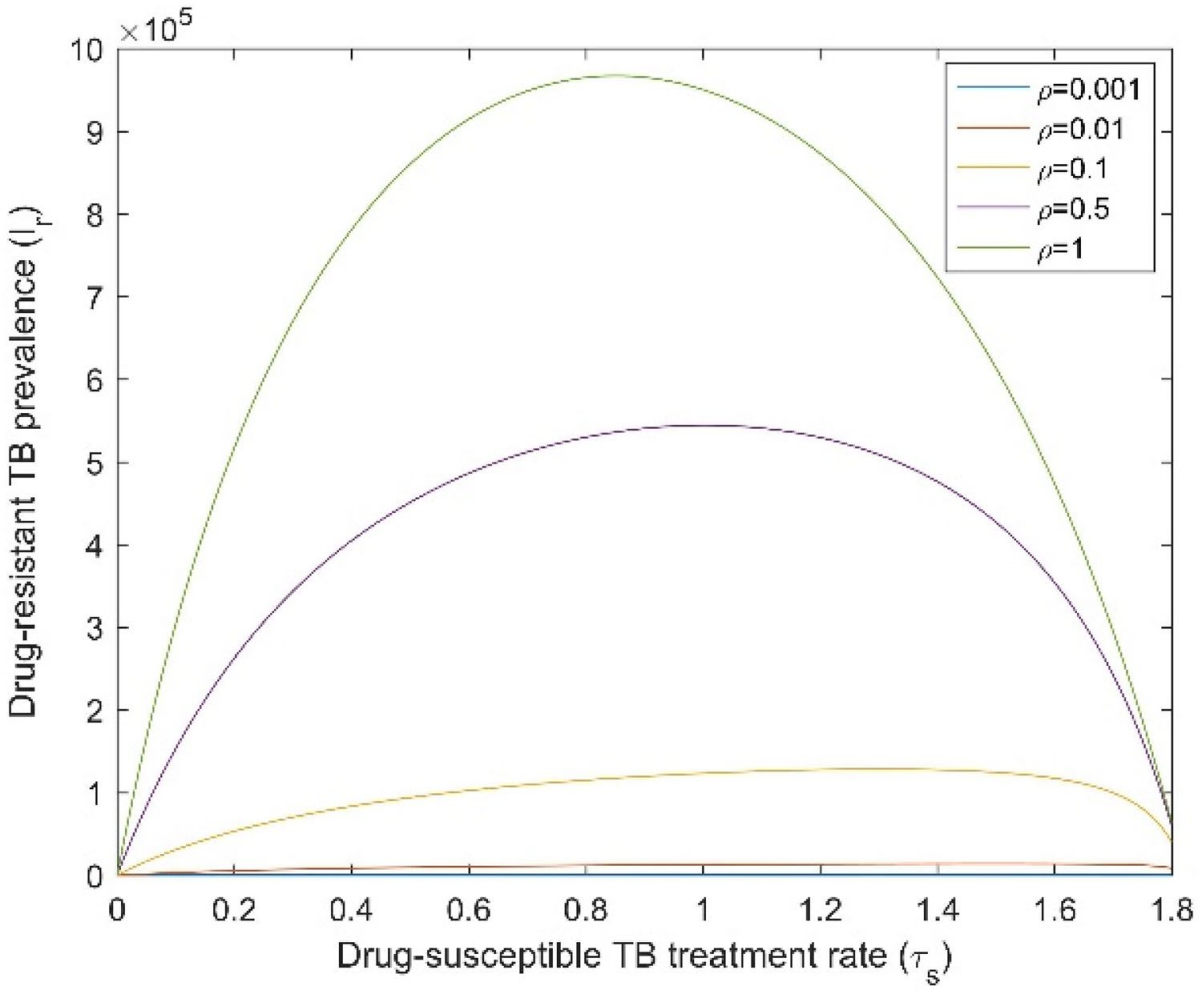
Effect of DS TB treatment rate (${\uptau }_{{\text{s}}}$) on DR TB prevalence when ${\upbeta }_{{\text{s}}}$ and ${\upbeta }_{{\text{r}}}$ are fixed. All remaining parameters consider their baseline values as reported in Table 1.

Figures 6 and 7 display the impact of the DS TB treatment rate $\left( {{\uptau }_{{\text{s}}} } \right)$ and amplification (${\uprho }$) on the total TB and DR TB prevalence, when both infectious rates $\left( {\upbeta _{{\text{s}}} ,\;\upbeta _{{\text{r}}} } \right)$ are fixed. When we increase the amplification, the total TB and DR TB prevalence also increases. Conversely, Fig. 7 shows that for high amplification, DR TB prevalence increased when the DS TB treatment rate increased from zero to around 0.8 to 0.9 and then dropped to a common point. For small amplification values, the DR TB proportion only raised up to the common point. This point is the DR TB-only equilibrium which becomes stable when the basic reproduction ratio of DR TB becomes higher than the effective reproduction number of DS TB and higher than one. Numerical analysis shows that for adequately high amplification, the DR TB prevalence will exceed that of its inherent equilibrium value when the DS TB is in existence and is being treated. From the above numerical analysis, it is clear that proper treatment is very important for DS TB patients otherwise it will lead to the creation of new cases of DR TB which can become the dominant strain.

**Figure 8:**
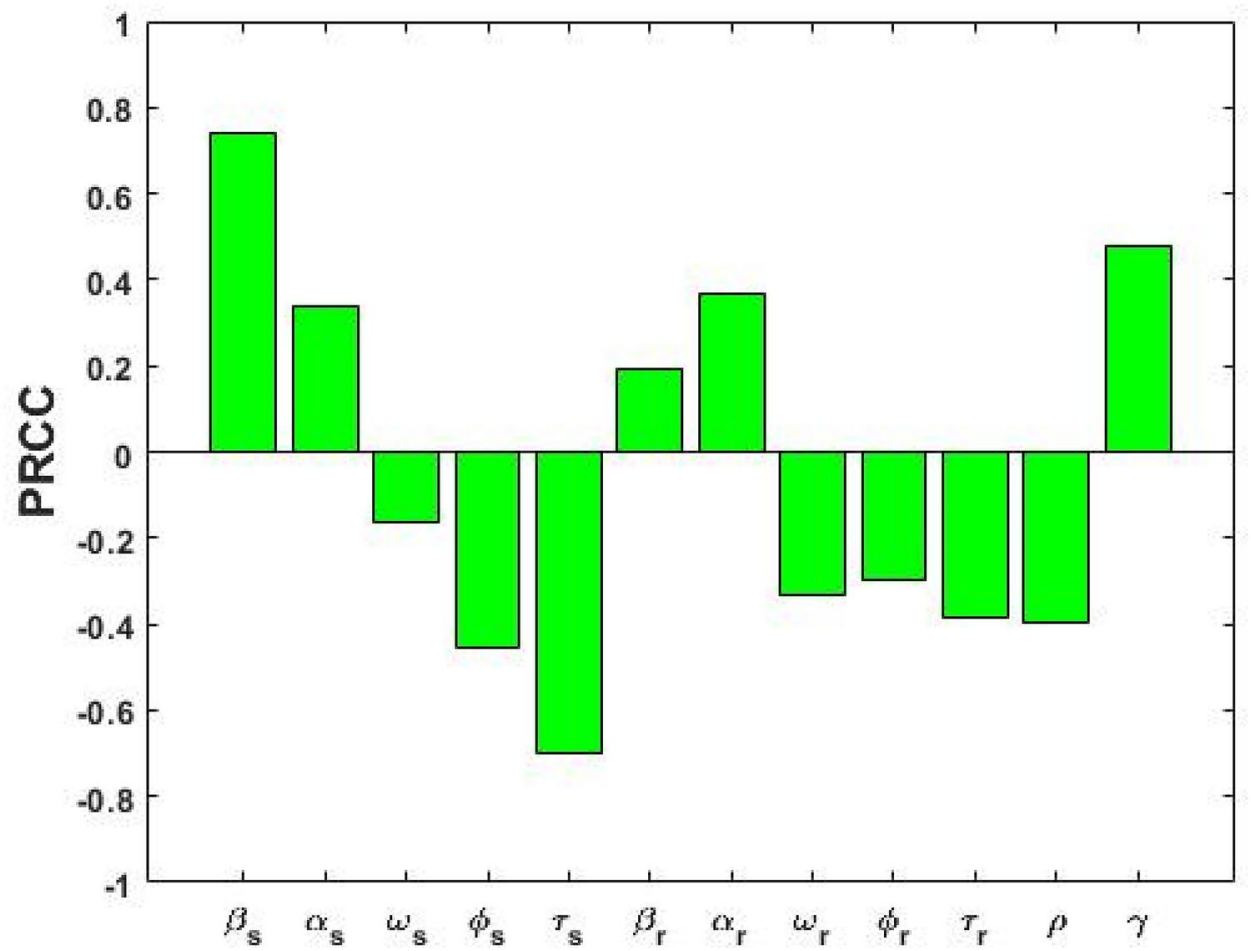
PRCC values representing the association between model output ${\text{I}}_{{\text{s}}}$ and the model parameters ${\upbeta }$ (transmission rate), ${\upalpha }$ (progression rate), ${\upomega }$ (recovery rate), $\phi$ (disease related death rate), ${\uptau }$ (treatment rate) and ${\uprho }$ (amplification rate), when ${\text{R}}_{{0{\text{s}}}} > {\text{max}}\left[ {{\text{R}}_{{0{\text{r}}}} ,{ }1} \right]$. Subscripts ${\text{s}}$ and ${\text{r}}$ denote DS and DR quantities, respectively.

We performed sensitivity analysis to explore the quantitative relationship between parameters and model outcomes including DS, DR and total TB prevalence. Figure 8 displays the association between the co-existent equilibrium value of DS TB prevalence (${\text{I}}_{{\text{s}}}$) and model parameters $\upbeta _{{\text{s}}} , \upalpha _{{\text{s}}} ,\upomega _{{\text{s}}} , \upphi _{{\text{s}}} ,\uptau _{{\text{s}}} , \upbeta _{{\text{r}}} , \upalpha _{{\text{r}}} ,\upomega _{{\text{r}}} , \upphi _{{\text{r}}} ,\uptau _{{\text{r}}} , \uprho$ and ${\upgamma }$, when ${\text{R}}_{{0{\text{s}}}} > {\text{max}}\left[ {{\text{R}}_{{0{\text{r}}}} ,{ }1} \right]$. From Fig. 8 it is observed that DS TB prevalence (${\text{I}}_{{\text{s}}}$) has a positive association with ${\upbeta }_{{\text{s}}} ,{{ \upalpha }}_{{\text{s}}} ,{{ \upbeta }}_{{\text{r}}} ,{{ \upalpha }}_{{\text{r}}}$, and ${{ \upgamma }}$, indicating that a positive change in any of these parameters will increase the DS TB prevalence (${\text{I}}_{{\text{s}}}$). We found that the association between the transmission rate ${\upbeta }_{{\text{s}}}$ and DS TB prevalence (${\text{I}}_{{\text{s}}}$) has the highest positive impact of all parameters, which is expected given the reproduction number is proportional to ${\upbeta }_{{\text{s}}}$. The public health lesson is that reducing exposure between infected and uninfected individuals is the most important public health intervention, dominating the importance of good treatment, reduced relapse, and amplification. Public health services should concentrate on those who are in close and prolonged contact with an infectious individual (i.e. individuals who are living in the same household with infectious TB cases) who currently have extensive exposure.

In contrast, parameters ${\upomega }_{{\text{s}}} ,\phi_{{\text{s}}} ,{\uptau }_{{\text{s}}} ,{{ \upomega }}_{{\text{r}}} ,{ }\phi_{{\text{r}}} ,{{ \uptau }}_{{\text{r}}} { }$ and ${\uprho }$ have a negative correlation with DS TB prevalence (${\text{I}}_{{\text{s}}}$), which means that increasing these parameters values will consequently decrease the ${\text{I}}_{{\text{s}}}$ prevalence. From the analysis, we also found that the treatment rate $\left( {{\uptau }_{{\text{s}}} } \right)$ is the second most important parameter and its association with DS TB prevalence. Our finding is consistent with previous studies^12,14,26^, and the WHO which recommended that early treatment and cure of infectious cases of TB to cut the chain of transmission of TB infection in the community. Therefore, quick identification of presumptive TB cases, rapid diagnosis, and early initiation of treatment and successful completion of treatment are the most effective ways of preventing TB. Further, amplification has a negative impact on DS TB prevalence because some of the DS TB-infected individuals move to the DR TB-infected state due to incorrect treatment.

**Figure 9:**
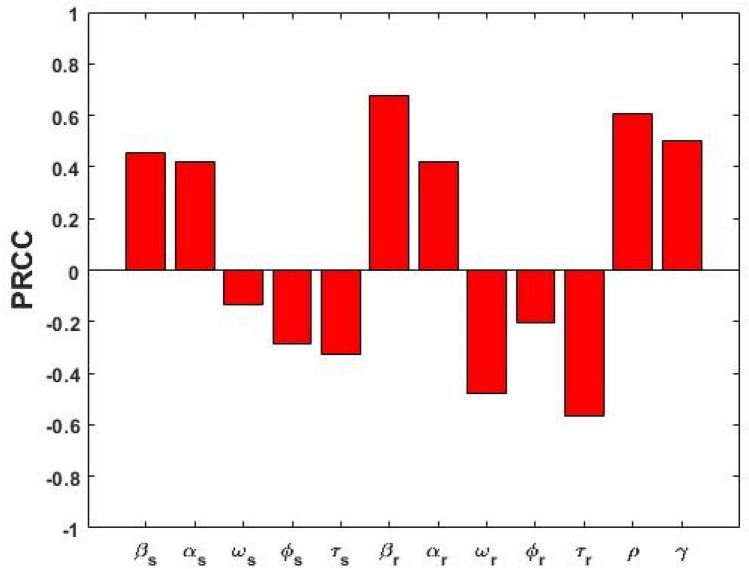
PRCC values showing the association between model output ${\text{I}}_{{\text{r}}}$ and the model parameters ${{ \upbeta }}_{{\text{s}}}$, ${\upalpha }_{{\text{s}}}$ ${\upomega }_{{\text{s}}}$, $\phi_{{\text{s}}} ,{{ \uptau }}_{{\text{s}}} ,{{ \upbeta }}_{{\text{r}}} ,{{ \upalpha }}_{{\text{r}}} ,{{ \upomega }}_{{\text{r}}} ,$ $\phi_{{\text{r}}} ,{{ \uptau }}_{{\text{r}}}\text{and} {{ \uprho }}$, when ${\text{R}}_{{0{\text{s}}}} > {\text{R}}_{{0{\text{r}}}} {\text{ and R}}_{{0{\text{s}}}} > 1$.

Figure 9 represents the correlation between the DR TB prevalence and corresponding model parameters ${\upbeta }_{{\text{s}}} ,{{ \upalpha }}_{{\text{s}}} ,{\upomega }_{{\text{s}}} ,\phi_{{\text{s}}} ,{\uptau }_{{\text{s}}} ,{{ \upbeta }}_{{\text{r}}} ,{{ \upalpha }}_{{\text{r}}} ,{\upomega }_{{\text{r}}} ,{ }\phi_{{\text{r}}} , {\uptau }_{{\text{r}}} ,{{ \uprho }}$ and ${\upgamma }$ when ${\text{R}}_{{0{\text{s}}}} > {\text{max}}\left[ {{\text{R}}_{{0{\text{r}}}} ,{ }1} \right]$. Parameters ${\upbeta }_{{\text{s}}}$, ${\upalpha }_{{\text{s}}} , {\upbeta }_{{\text{r}}} , {\upalpha }_{{\text{r}}}$, ${\uprho }$ and ${\upgamma }$ have positive PRCC values and parameters ${{ \upomega }}_{{\text{s}}} ,\phi_{{\text{s}}} ,{{ \uptau }}_{{\text{s}}} ,{\upomega }_{{\text{r}}} ,$ $\phi_{{\text{r}}}$ and ${\uptau }_{{\text{r}}}$ have negative PRCC values. Our finding is in line with observations and shows that amplification $\left( {\uprho } \right)$ has a positive correlation with DR TB prevalence at the co-existent equilibrium because it is the pathway from DS TB to DR TB.

We further performed sensitivity analysis for the total TB prevalence $\left( {{\text{I}}_{{\text{s}}} + {\text{I}}_{{\text{r}}} } \right)$ and DR TB prevalence according to their corresponding model parameters when ${\text{R}}_{{0{\text{s}}}} > {\text{max}}\left[ {{\text{R}}_{{0{\text{r}}}} ,{ }1} \right]$ and ${\text{R}}_{{0{\text{r}}}} > {\text{max}}\left[ {{\text{R}}_{{0{\text{s}}}} ,1} \right]$ respectively (see supplementary materials: sensitivity analysis section, Figure S4 and S5).

The sensitivity indices of ${\text{R}}_{{0{\text{s}}}}$ and ${\text{R}}_{{0{\text{r}}}}$ with respect to each parameter are given in Table 2. In the sensitivity indices of ${\text{R}}_{{0{\text{s}}}}$ and ${\text{ R}}_{{0{\text{r}}}}$, the most significant parameters are the transmission rates of DS TB,${{ \upbeta }}_{{\text{s}}}$ and DR TB,${{ \upbeta }}_{{\text{r}}}$. Since ${{ \Upsilon }}_{{{\upbeta }_{{\text{s}}} }}^{{{\text{R}}_{{0{\text{s}}}} }} = 1$, and ${{ \Upsilon }}_{{{\upbeta }_{{\text{r}}} }}^{{{\text{R}}_{{0{\text{r}}}} }} = 1$, increasing (or decreasing) the transmission rates, ${\upbeta }_{{\text{s}}}$ and ${\upbeta }_{{\text{r}}}$ of DS TB and DR TB by a particular percentage, increases (or decreases) the reproduction numbers ${\text{R}}_{{0{\text{s}}}}$ and ${\text{R}}_{{0{\text{r}}}}$ by the same percentage.

**Table 2:** Sensitivity indices for ${\text{R}}_{{0{\text{s}}}}$ and ${\text{R}}_{{0{\text{r}}}}$.

Parameter	Sensitivity index (${\text{R}}_{{0{\text{s}}}}$)	Parameter	Sensitivity index (${\text{R}}_{{0{\text{r}}}}$)
${\upbeta }_{{\text{s}}}$	+ 1.000	${\upbeta }_{{\text{r}}}$	+ 1.000
${\upalpha }_{{\text{s}}}$	+ 0.100	${\upalpha }_{{\text{r}}}$	+ 0.100
${\upomega }_{{\text{s}}}$	− 0.178	${\upomega }_{{\text{r}}}$	− 0.093
$\phi_{{\text{s}}}$	− 0.229	$\phi_{{\text{r}}}$	− 0.288
${\uptau }_{{\text{s}}}$	− 0.583	${\uptau }_{{\text{r}}}$	− 0.607

## Discussion and conclusion

In this paper, we formulated and analysed a novel two-strain TB model with amplification: one strain for DS TB; and another for DR TB. Here, we considered amplification as the process by which an individual infected with DS TB develops infection with a resistant strain of TB, reflecting treatment failure for individuals on first line drug therapy.

We found three equilibrium points of our proposed model; the disease-free equilibrium; the mono-existent equilibrium, when DR TB dominated in this system; and the co-existent equilibrium, when DS TB dominated in this system. The next generation matrix method was used to calculate the basic reproduction number of the different TB strains, denoted by ${\text{R}}_{{0{\text{s}}}}$ for DS TB and ${\text{R}}_{{0{\text{r}}}}$ for DR TB. The value of the basic reproduction numbers, namely ${\text{R}}_{{0{\text{s}}}}$ and ${\text{R}}_{{0{\text{r}}}}$, and biological parameters of the model, were estimated on the basis of available data and are tabulated in Table 1. Furthermore, dynamical system analyses were also used to investigate the local stability of the disease-free equilibrium and mono-existent equilibrium. This analysis showed that stability depends on the threshold quantities, i.e. the basic reproduction numbers ${\text{R}}_{{0{\text{s}}}}$ and ${\text{R}}_{{0{\text{r}}}}$. If $\max \left[ {{\text{R}}_{{0{\text{s}}}} ,{\text{R}}_{{0{\text{r}}}} } \right] < 1$, the disease-free equilibrium is globally asymptotically stable, which means that the disease naturally dies out. If ${\text{R}}_{{0{\text{r}}}} > {\text{max}}\left[ {{\text{R}}_{{0{\text{s}}}} ,1} \right]$, DS TB dies out but DR TB persists in the population. If ${\text{R}}_{{0{\text{s}}}} > {\text{max}}\left[ {{\text{R}}_{{0{\text{r}}}} ,1} \right]$, then DS TB and DR TB both persist in the population. This analysis can help us to identify regions in the parameter space where the various asymptotic states are stable or unstable, thus allowing us to predict the long-term behaviour of the DS and DR TB dynamics. This information can advise the Bangladesh National TB Control Program to reduce the period of infectiousness down until $\max \left[ {{\text{R}}_{{0{\text{s}}}} ,{\text{R}}_{{0{\text{r}}}} } \right] < 1$.

The existence and stability of the transmission dynamics of sensitive and resistant TB bacteria within and between-hosts models were considered in previous modelling studies^27,28^. Previous studies showed that if the basic reproduction numbers of sensitive bacteria and resistant bacteria is less than one then the bacteria are cleared. If the basic reproduction number of resistant bacteria is greater than one but that of the sensitive bacteria is less than one then only resistant bacteria persist. Finally, the previous studies also showed that both sensitive and resistant bacteria persist if the sensitive bacteria’s basic reproduction number is greater than one and the resistant bacteria’s basic reproduction number bigger than certain threshold but less than one^27,28^, which is not the same as our results, which do not require the basic reproduction number of the resistant bacteria to be above a threshold in order to achieve co-existance, owing to the amplification pathway.

Several TB modelling studies^14,17^ explored the impact of single and combination intervention strategies. Results show that combination intervention strategies are the most effective for reducing the burden of TB. In this study, we considered a Bangladesh-specific six-compartmental two-strain SLIRS model with amplification. An important feature of this model is the coupling between the two strains representing the flow of infected individuals who acquire resistance during treatment. We assumed amplification develops mainly through the choice of naturally occurring mutations in the presence of inappropriate treatment. We also considered a natural recovery parameter and treatment parameter which are not considered in the earlier analyses^14,17^. Here, we additionally allow recovered people to move to the susceptible class due to loss of immunity, a feature that is also not considered in previous studies^14,17,18^.

Our model determined that from the explicit formulae for ${\text{R}}_{{0{\text{s}}}}$ and ${\text{R}}_{{0{\text{r}}}}$, it is clear that these basic reproduction numbers depend on transmission rates ${\upbeta }_{{\text{s}}} { }\left( {{\upbeta }_{{\text{r}}} } \right)$, progression rates ${\upalpha }_{{\text{s}}} \left( {{\upalpha }_{{\text{r}}} } \right)$, recovery rates ${\upomega }_{{\text{s}}} { }\left( {{\upomega }_{{\text{r}}} } \right)$, disease related death rates $\phi_{{\text{s}}} { }\left( {\phi_{{\text{r}}} } \right)$, and treatment rates ${\uptau }_{{\text{s}}} { }\left( {{\uptau }_{{\text{r}}} } \right)$. From the sensitivity analysis it is also clear that the most important parameters are the transmission rates ${\upbeta }_{{\text{s}}} { }\left( {{\upbeta }_{{\text{r}}} } \right)$ followed by the treatment rates ${\uptau }_{{\text{s}}} { }\left( {{\uptau }_{{\text{r}}} } \right)$. Therefore, to control and eradicate DS TB and DR TB infection, it is important to consider the following strategies: the first and most important strategy is to minimize the contact rates ${\upbeta }_{{\text{s}}} { }\left( {{\upbeta }_{{\text{r}}} } \right)$ with infected individuals. There are many ways we can minimize the contact rates, such as personal respiratory protection; this includes wearing of masks by patients to minimise dispersal of TB bacilli when they talk, cough, yawn or sneeze. In addition, basic infection control measures have to be taught to patients such as covering the nose and mouth during coughing and sneezing and to discard used tissues into covered bins. Environmental control measures maximise dilution and air exchange and decontaminate air when adequate ventilation cannot be reached in high-risk areas. Any ventilation system must be monitored and maintained on a regular schedule. Diagnosis campaigns are also needed to control transmission.

The second-most important strategy is to increase the treatment rates ${\uptau }_{{\text{s}}} { }\left( {{\uptau }_{{\text{r}}} } \right)$ of infective individuals. Moreover, higher values of amplification $\left( {\uprho } \right)$ when combined with high ${\uptau }_{{\text{s}}}$, lead to higher prevalence of DR TB—simulating inadequate or poorly administered treatment resulting in the creation of new cases of DR TB. Therefore, we suggest that for problematic DR TB, measures of the reproduction numbers of the DS and DR TB be performed together with the risk of amplification, to assure optimal levels of treatment be utilized to reduce the risk of DR TB. Well-administered first-line treatment for DS TB is the best way to prevent acquisition of resistance. Timely identification of DR TB and adequate treatment regimens with second-line drugs administered are essential to prevent rises in DR TB prevalence. However, in developing countries (e.g. Bangladesh) it is very difficult to isolate infectious individuals due to the high cost of long-term treatment. Therefore, we propose the most feasible and optimal strategy to eliminate DS TB and DR TB in Bangladesh is to increase the treatment rates by decreasing the treatment cost allowing universal health care.

## Supplementary Information

Supplementary Information.

## Data Availability

The datasets produced during the study are available from the corresponding author on reasonable request.
