# Supplementary material for: Mathematical analysis of a two-strain tuberculosis model in Bangladesh
Source: Sci Rep. 2022 Mar 7;12:3634. doi: 10.1038/s41598-022-07536-2 (PMC8901732; doi:10.1038/s41598-022-07536-2)
Supplement: Supplementary file 1 — Supplementary Information. [file 41598_2022_7536_MOESM1_ESM.docx]

Supplementary materials to “Mathematical analysis of a two-strain tuberculosis model in Bangladesh”

**Model equations**

$\frac{\mathrm{dS}}{\mathrm{dt}}=\mu N-\beta_{s}I_{s}S-\beta_{r}I_{r}S-\mu S+\gamma R+\phi_{s}I_{s}+\phi_{r}I_{r}$, (S1)

$\frac{dL_{s}}{\mathrm{dt}}=\beta_{s}I_{s}S-\alpha_{s}L_{s}-\mu L_{s}$, (S2)

$\frac{dI_{s}}{\mathrm{dt}}=\alpha_{s}L_{s}-\omega_{s}I_{s}-\mu I_{s}-\tau_{s}I_{s}-\phi_{s}I_{s}$, (S3)

$\frac{dL_{r}}{\mathrm{dt}}=\beta_{r}I_{r}S-\alpha_{r}L_{r}-\mu L_{r}$, (S4)

$\frac{dI_{r}}{\mathrm{dt}}=\alpha_{r}L_{r}-\omega_{r}I_{r}-\mu I_{r}+\rho\tau_{s}I_{s}-\phi_{r}I_{r}-\tau_{r}I_{r}$, (S5)

$\frac{\mathrm{dR}}{\mathrm{dt}}=\omega_{s}I_{s}+\omega_{r}I_{r}-\gamma R-\mu R+\left( 1-\rho\right)\tau_{s}I_{s}+\tau_{r}I_{r}$. (S6)

where

$N\left( t \right)=S\left( t \right)+L_{s}\left( t \right)+I_{s}\left( t \right)+L_{r}\left( t \right)+I_{r}\left( t \right)+R\left( t \right)$. (S7)

**Estimation of model parameters**

The objective function used in the parameter estimation for the annual incidence is as follows

$\hat{\theta}=argmin\sum_{i=1}^{n} \left( \int_{t_{i}}^{t_{i}+1} \left( \alpha_{s}L_{s}(t^{'}) \right)\mathrm{dt}^{'}-{\mathrm{data}_{t}}_{\mathrm{ip}} \right)^{2}$, and

$\hat{\theta_{1}}=argmin\sum_{i=1}^{n} \left( \int_{t_{i}}^{t_{i}+1} \left( {{\rho\tau}_{s}I}_{s}(t^{'})+\alpha_{r}L_{r}(t^{'}) \right)dt^{'}-{\mathrm{data}_{t}}_{\mathrm{iq}} \right)^{2}$

where $\mathrm{dat}a_{t_{\mathrm{ip}}}$ and $\mathrm{dat}a_{t_{\mathrm{iq}}}$ denotes the actual DS and DR TB incidence data respectively and $\alpha_{s}L_{s}$ and $\left( {{\rho\tau}_{s}I}_{s}+{\alpha_{r}L}_{r} \right)$ are the corresponding model incidence solutions at time $t_{i}$ respectively. n is the number of available data points. The associated state variable and parameters of the model (S1) – (S6) are tabulated in Table 1.

**Existence of equilibria**

Three types of equilibrium solutions appear in this system: the disease-free equilibrium, which is reached when both basic reproduction numbers are less than one i.e. $\max\left[ R_{0s},R_{0r} \right]<1$; the mono-existent endemic equilibrium, which is reached when the basic reproduction number of DR TB is greater than the basic reproduction number of DS TB and one i.e. $R_{0r}>\max\left[ R_{0s},1 \right]$; and the co-existent endemic equilibrium, which is reached when the basic reproduction number of DS TB is greater than that of DR TB and one i.e. $R_{0s}>max[R_{0r},1]$. We discuss these in order below.

Clearly, equations (S1) – (S6) always have an infection-free equilibrium

$E^{*}=\left( S^{*}, {L_{s}^{*}, I}_{s}^{*}, L_{r}^{*}, I_{r}^{*}, R^{*} \right)=\left( N, 0, 0, 0, 0, 0 \right).$

From equations (S1) - (S6) we can calculate the mono-existent endemic equilibrium point

$E^{^}=(S^{^}, 0, 0,L_{r}^{^}, I_{r}^{^},R^{^})$ at which DR TB persists and DS TB dies out:

$S^{^}=\frac{N}{R_{0r}}$,

$$L_{s}^{^}=0,$$

$$I_{s}^{^}=0,$$

$I_{r}^{^}=\frac{\mu}{\beta_{r}}\frac{\left( R_{0r}-1 \right)}{\sigma}$ ,

$L_{r}^{^}=\frac{\chi_{r}}{\alpha_{r}} I_{r}^{^}$ ,

$R^{^}=\frac{(\tau_{r}+\omega_{r})}{(\gamma+\mu)}I_{r}^{^}$, (S8)

where $\sigma=\frac{\left( \gamma\left( 1-\frac{\alpha_{r}\left( \omega_{r}+\phi_{r}+\tau_{r} \right)}{\left( \alpha_{r}+\mu\right)\left( \omega_{r}+\phi_{r}+\tau_{r}+\mu\right)} \right)+\mu\left( 1-\frac{\alpha_{r}\phi_{r}}{\left( \alpha_{r}+\mu\right)\chi_{r}} \right) \right)}{\gamma+\mu}\Rightarrow0<\sigma<1$. From (S8) if $R_{0r}>1$, equations (S1) – (S6) have a unique boundary equilibrium $E^{^}=(S^{^}, 0, 0,L_{r}^{^}, I_{r}^{^},R^{^})\in D$.

Finally, we can examine the co-existent endemic equilibrium of the model equations. If the co-existent endemic equilibrium is $E^{\dagger}=(S^{\dagger}, L_{s}^{\dagger}, I_{s}^{\dagger}, L_{r}^{\dagger}, I_{r}^{\dagger}, R^{\dagger})$, from equations (S1)—(S6), we obtain

$S^{\dagger}=\frac{N}{R_{0s}}$,

$L_{s}^{\dagger}= \frac{\chi_{s}}{\alpha_{s}} I_{s}^{\dagger}$ ,

$L_{r}^{\dagger}=\frac{\rho\tau_{s}R_{0r}}{\alpha_{r}} I_{s}^{\dagger}$,

$I_{r}^{\dagger}= \frac{\rho R_{0s}\tau_{s}}{\chi_{r}(R_{0s}-R_{0r})}I_{s}^{\dagger}$,

$R^{\dagger}=\frac{1}{(\gamma+\mu)}\left( (\left( 1-\rho\right)\tau_{s}+\omega_{s})+\frac{\rho\tau_{s}R_{0s}\left( \omega_{r}+\tau_{r} \right)}{\chi_{r}(R_{0s}-R_{0r})} \right)I_{s}^{\dagger}$, (S9)

To check if $E^{\dagger}\in D$, it remains to determine the sign of state variable $I_{s}^{\dagger}$. This is most easily done using the total population conservation equation:

$N=S^{\dagger}+L_{s}^{\dagger}+I_{s}^{\dagger}+L_{r}^{\dagger}+I_{r}^{\dagger}+R^{\dagger}$,

$I_{s}^{\dagger}=N\left( 1-\frac{1}{R_{0s}} \right)\eta$ (S10)

where $\eta=\left( 1+\frac{\chi_{s}}{\alpha_{s}}+\frac{\rho\tau_{s}R_{0r}}{\alpha_{r}}+\frac{\rho R_{0s}\tau_{s}}{\chi_{r}\left( R_{0s}-R_{0r} \right)}+\frac{\left( 1-\rho\right)\tau_{s}+\omega_{s}}{\left( \gamma+\mu\right)}+\frac{\left( \omega_{r}+\tau_{r} \right)}{\left( \gamma+\mu\right)} \frac{\rho R_{0s}\tau_{s}}{\chi_{r}\left( R_{0s}-R_{0r} \right)} \right)^{-1}>0$ for $R_{0s}>R_{0r}$. We then have

$\mathrm{sign}\left( I_{s}^{\dagger} \right)=sign\left( 1-\frac{1}{R_{0s}} \right)$ $\Rightarrow R_{0s}>1\Rightarrow I_{s}^{\dagger}>0$.

From (S9), for the co-existent region in which the condition $I_{s}^{\dagger}>0$ is required, a necessary and sufficient condition for the endemic population $I_{s}^{\dagger}$ to be non-negative is to have both $R_{0s}>1$ and $R_{0s}>R_{0r}$. That is, if $R_{0s}>max[R_{0r}, 1]$ then (S1) – (S6) have a co-existent endemic equilibrium$E^{\dagger}=\left( S^{\dagger}, L_{s}^{\dagger}, I_{s}^{\dagger}, L_{r}^{\dagger}, I_{r}^{\dagger}, R^{\dagger} \right)\in D$.

**Stability analysis**

To examine the stability of the equilibria of equations (S1) – (S6) the following outcomes are proven.

***Disease-free equilibrium:***

***Lemma 1:*** The disease-free equilibrium of the model is locally and globally asymptotically stable if $max[R_{0s},R_{0r}]<1$ and unstable if $\max\left[ R_{0s},R_{0r} \right]>1$.

***Proof:*** The Jacobian matrix of the system (S1) – (S6) which is given by

$$J=\left( {\begin{aligned} -({\beta_{s}I_{s}+\beta}_{r}I_{r}+\mu) \\ \beta_{s}I_{s} \\ 0 \\ \beta_{r}I_{r} \\ 0 \\ 0 \end{aligned}}\begin{aligned} 0 \\ -(\alpha_{s}+\mu) \\ \alpha_{s} \\ 0 \\ 0 \\ 0 \end{aligned}\begin{aligned} -\beta_{s}S+\phi_{s} \\ \beta_{s}S \\ -(\omega_{s}+\phi_{s}+\tau_{s}+\mu) \\ 0 \\ \rho\tau_{s} \\ \left( 1-\rho\right)\tau_{s}+\omega_{s} \end{aligned}\begin{aligned} 0 \\ 0 \\ 0 \\ -(\alpha_{r}+\mu) \\ \alpha_{r} \\ 0 \end{aligned}\begin{aligned} -\beta_{r}S+\phi_{r} \\ 0 \\ 0 \\ \beta_{r}S \\ -(\omega_{r}+\phi_{r}+\tau_{r}+\mu) \\ (\omega_{r}+\tau_{r}) \end{aligned}\begin{aligned} \gamma\\ 0 \\ 0 \\ 0 \\ 0 \\ -(\gamma+\mu) \end{aligned} \right)$$

at the disease-free equilibrium point, $E^{*}$, reduces to

$$J^{*}=\left( {\begin{aligned} -\mu\\ 0 \\ 0 \\ 0 \\ 0 \\ 0 \end{aligned}}\begin{aligned} 0 \\ -(\alpha_{s}+\mu) \\ \alpha_{s} \\ 0 \\ 0 \\ 0 \end{aligned}\begin{aligned} -\beta_{s}N+\phi_{s} \\ \beta_{s}N \\ -(\omega_{s}+\phi_{s}+\tau_{s}+\mu) \\ 0 \\ \rho\tau_{s} \\ \left( 1-\rho\right)\tau_{s}+\omega_{s} \end{aligned}\begin{aligned} 0 \\ 0 \\ 0 \\ -(\alpha_{r}+\mu) \\ \alpha_{r} \\ 0 \end{aligned}\begin{aligned} -\beta_{r}N+\phi_{r} \\ 0 \\ 0 \\ \beta_{r}N \\ -(\omega_{r}+\phi_{r}+\tau_{r}+\mu) \\ (\omega_{r}+\tau_{r}) \end{aligned}\begin{aligned} \gamma\\ 0 \\ 0 \\ 0 \\ 0 \\ -(\gamma+\mu) \end{aligned} \right).$$

The construction of the Jacobian matrix $J^{*}$ permits us to immediately read off two eigenvalues,$\lambda_{1}=-\mu and \lambda_{2}=-\left( \gamma+\mu\right)$. The remaining four eigenvalues can be obtained from the following reduced matrix

$\bar{J}^{*}=\left( \begin{aligned} -(\alpha_{s}+\mu) \\ \alpha_{s} \\ 0 \\ 0 \end{aligned}\begin{aligned} \beta_{s}N \\ -(\omega_{s}+\phi_{s}+\tau_{s}+\mu) \\ 0 \\ \rho\tau_{s} \end{aligned}\begin{aligned} 0 \\ 0 \\ -(\alpha_{r}+\mu) \\ \alpha_{r} \end{aligned}\begin{aligned} 0 \\ 0 \\ \beta_{r}N \\ -(\omega_{r}+\phi_{r}+\tau_{r}+\mu) \end{aligned} \right).$

This matrix can be written in block form as

$\bar{J}^{*}= \left( \begin{matrix} A & B \\ C & D \end{matrix} \right)$

where, $A=\left( \begin{matrix} -\alpha_{s}-\mu& \beta_{s}N \\ \alpha_{s} & -\omega_{s}-\varphi_{s}-\tau_{s}-\mu\end{matrix} \right), B=\left( \begin{matrix} 0 & 0 \\ 0 & 0 \end{matrix} \right), C=\left( \begin{matrix} 0 & 0 \\ 0 & \rho\tau_{s} \end{matrix} \right)$and

$D=\left( \begin{matrix} -\alpha_{r}-\mu& \beta_{r}N \\ \alpha_{r} & -\omega_{r}-\phi_{r}-\tau_{r}-\mu\end{matrix} \right)$.

Here the matrix $A$ corresponds to DS TB dynamics, the matrix $D$ represents DR TB dynamics, and matrix $C$ gives the flow between DS and DR TB.

The characteristic equation of the two-by-two block matrix$\bar{J}^{*}$ is

$\det\left( A-\lambda I \right)\det\left( (D-\lambda I)-C{(A-\lambda I)}^{-1}B \right)=0$.

Since $B=\left( \begin{matrix} 0 & 0 \\ 0 & 0 \end{matrix} \right)$ this reduces to

$\det\left( A-\lambda I \right)\det\left( D-\lambda I \right)=0$.

This relation implies that we can apply the Routh-Hurwitz criteria ^23^ (It is a mathematical test that is necessary and sufficient condition for the stability of a linear time invariant system) for stability to matrices $A$ and $D$ directly, and independently. We then have

$\mathrm{trace}\left( A \right)=-\left( \alpha_{s}+\mu\right)+\left( -\omega_{s}-\phi_{s}-\tau_{s}-\mu\right)<0$,

and

$\det\left( A \right)=\left( \alpha_{s}+\mu\right)\left( \omega_{s}+\phi_{s}+\tau_{s}+\mu\right)-\alpha_{s}\beta_{s}N>0$,

which we can reformulate as

$R_{0s}<1$. (S11)

Now for DR TB cases (i.e. matrix $D$)

$\mathrm{trace}\left( D \right)=-\left( \alpha_{r}+\mu\right)+\left( -\omega_{r}-\phi_{r}-\tau_{r}-\mu\right)<0$,

and

$\det\left( D \right)=\left( \alpha_{r}+\mu\right)\left( \omega_{r}+\phi_{r}+\tau_{r}+\mu\right)-\alpha_{r}\beta_{r}N>0$,

which can similarly be rewritten as

$R_{0r}<1$. (S12)

Therefore, when$R_{0s}<1$ and $R_{0r}<1$, the disease-free equilibrium $E^{*}$of (S11) and (S12) is locally asymptotically stable. However, when $R_{0s}>1$ or $R_{0r}>1$, at least one of the roots of the characteristic equation has a positive real part and the disease-free equilibrium point is unstable.

Now we can examine the global stability of the disease-free equilibrium $E^{*}$ when $R_{0s}<1$ and $R_{0r}<1$. To do this, we first show that the infected subpopulations $L_{s}$ and $I_{s}$ approach zero for $R_{0s}<1$ using an appropriate Lyapunov function ^24^ (It is a scalar function that is used to prove the stability of an equilibrium of an ordinary differential equation):

$V_{s}(t)=L_{S}(t)+\frac{\left( \alpha_{s}+\mu\right)}{\alpha_{s}}I_{s}(t)$.

Now taking the derivative of $V_{s}(t)$, we have

$\dot{V}_{S}=\dot{L}_{s}+\frac{\left( \alpha_{S}+\mu\right)}{\alpha_{S}}\dot{I}_{s}$,

$=\beta_{s}I_{s}S-\left( \alpha_{s}+\mu\right)L_{s}+\left( \alpha_{s}+\mu\right)L_{s}-\frac{\chi_{s}\left( \alpha_{s}+\mu\right)}{\alpha_{s}}I_{s}$,

$=\beta_{s}I_{s}S-\frac{\chi_{s}\left( \alpha_{s}+\mu\right)}{\alpha_{s}}I_{s}$,

$= \frac{\chi_{s}\left( \alpha_{s}+\mu\right)}{\alpha_{s}} \left( \frac{\alpha_{s}\beta_{s}S}{\left( \alpha_{s}+\mu\right)\chi_{s}}-1 \right)I_{s}$,

$\leq\frac{\chi_{s}\left( \alpha_{s}+\mu\right)}{\alpha_{s}} \left( R_{0s}-1 \right)I_{s}(t)$

where in the last line we have invoked the inequality $S\leq N$. It shows that if $R_{0s}<1$ we have $L_{s}\left( t \right),I_{s}(t)\to0$ as $t\to\infty$. Hence the hyperplane ${L_{s}=I}_{s}=0$ attracts all solutions of (S1) – (S6) whenever $R_{0s}<1$.

Since ${L_{s}\left( t \right)=I}_{s}(t)\to0$ as $t\to\infty$ for $R_{0s}<1$, it shows that $\rho\omega_{s}I_{s}\to0$, such that equation (S5) reduces to

$\dot{I}_{r}=\alpha_{r}L_{r}-\chi_{r}I_{r}$ .

In the same ways for DR TB ($L_{r}$ and$I_{r}$) as we used above for DS TB ($L_{s}$ and$I_{s}$) and introducing the Lyapunov function

$V_{r}\left( t \right)=L_{r}\left( t \right)+I_{r}(t)$

yields

$\dot{V}_{r}(t)$ $\leq\frac{\chi_{r}\left( \alpha_{r}+\mu\right)}{\alpha_{r}} \left( R_{0r}-1 \right)I_{r}(t)$.

Therefore if $R_{0r}<1$ we have ${L_{r}\left( t \right), I}_{r}(t)\to0$ as $t\to\infty$ and the hyperplane ${L_{r}=I}_{r}=0$ attracts all solutions of (S1) – (S6). It follows then that $R\to0$ and $S\to N$ such that $E^{*}$ is globally asymptotically stable when$\max\left[ R_{0s}, R_{0r} \right]<1$.

Epidemiologically it can be implied that TB can be eliminated from the community when both the basic reproduction numbers are less than one, i.e.$max[R_{0s},R_{0r}]<1$. If $max[R_{0s},R_{0r}]<1$ then this means the average number of infected individual develops less than one new infected individual over the period of infection and the infection dies out.

***Mono-existent endemic equilibrium***

***Lemma 2:*** If the mono-existent endemic equilibrium $E^{^}=(S^{^}, 0, 0,L_{r}^{^}, I_{r}^{^},R^{^})$ of the

equations (S1)—(S6) exists and$R_{0r}>{max[1,R}_{0s}]$ then $E^{^}$is locally asymptotically stable.

***Proof:*** For simplicity, we use the condition $R=N-S-L_{s}-I_{s}-L_{r}-I_{r}$ to eliminate the $R$ equation from the full system given in (S1) and consider the Jacobian matrix of the equations (S1)—(S5) at $E^{^},$which is given by

$J^{^}=\left( {\begin{aligned} -(\beta_{r}I_{r}^{^}+\mu+\gamma) \\ 0 \\ 0 \\ \beta_{r}I_{r}^{^} \\ 0 \end{aligned}}\begin{aligned} -\gamma\\ -(\alpha_{s}+\mu) \\ \alpha_{s} \\ 0 \\ 0 \end{aligned}\begin{aligned} -\beta_{s}S^{^}+\phi_{s}-\gamma\\ \beta_{s}S^{^} \\ -(\omega_{s}+\phi_{s}+\tau_{s}+\mu) \\ 0 \\ \rho\tau_{s} \end{aligned}\begin{aligned} -\gamma\\ 0 \\ 0 \\ -(\alpha_{r}+\mu) \\ \alpha_{r} \end{aligned}\begin{aligned} -\beta_{r}S^{^}+\phi_{r}-\gamma\\ 0 \\ 0 \\ \beta_{r}S^{^} \\ -(\omega_{r}+\phi_{r}+\tau_{r}+\mu) \end{aligned} \right)$.

Simultaneously interchanging rows and columns of the matrix $J^{^}$ we obtain the equivalent Jacobian

$$J^{^}=\left( {\begin{aligned} -(\beta_{r}I_{r}^{^}+\mu+\gamma) \\ \beta_{r}I_{r}^{^} \\ 0 \\ 0 \\ 0 \end{aligned}}\begin{aligned} -\gamma\\ -(\alpha_{r}+\mu) \\ \alpha_{r} \\ 0 \\ 0 \end{aligned}\begin{aligned} -\beta_{r}S^{^}+\phi_{r}-\gamma\\ \beta_{r}S^{^} \\ -(\omega_{r}+\phi_{r}+\tau_{r}+\mu) \\ 0 \\ 0 \end{aligned}\begin{aligned} -\gamma\\ 0 \\ 0 \\ -(\alpha_{s}+\mu) \\ \alpha_{s} \end{aligned}\begin{aligned} -\beta_{s}S^{^}+\phi_{s}-\gamma\\ 0 \\ \rho\tau_{s} \\ \beta_{s}S^{^} \\ -(\omega_{s}+\phi_{s}+\tau_{s}+\mu) \end{aligned} \right)$$

which can be written in block form as

$J^{^}=\left( \begin{matrix} A_{1} & A_{2} \\ A_{3} & A_{4} \end{matrix} \right)$

where$A_{1}=\left( \begin{matrix} {-(\beta}_{r}I_{r}^{^}+\mu+\gamma) & -\gamma& -\beta_{r}S^{^}+\phi_{r}-\gamma\\ \beta_{r}I_{r}^{^} & {-(\alpha}_{r}+\mu) & \beta_{r}S^{^} \\ 0 & \alpha_{r} & -(\omega_{r}+\phi_{r}+\tau_{r}+\mu) \end{matrix} \right)$,

$A_{2}=\left( \begin{matrix} -\gamma& -\beta_{s}S^{^}+\phi_{s}-\mu\\ 0 & 0 \\ 0 & \rho\tau_{s} \end{matrix} \right)$, $A_{3}=\left( \begin{matrix} 0 & 0 & 0 \\ 0 & 0 & 0 \end{matrix} \right)$, $A_{4}=\left( \begin{matrix} -(\alpha_{s}+\mu) & \beta_{s}S^{^} \\ \alpha_{s} & -(\omega_{s}+\phi_{s}+\tau_{s}+\mu) \end{matrix} \right)$.

The characteristic equation of$J^{^}$ is

$\det\left( J^{^}-\lambda I \right)=det\left( \begin{matrix} A_{1}-\lambda I & A_{2} \\ A_{3} & A_{4}-\lambda I \end{matrix} \right)=0$,

$\Rightarrow det (A_{1}-\lambda I)\det({(A}_{4}-\lambda I)-A_{3}{{(A_{1}-\lambda I)}^{-1}A}_{2})=0$ ,

Since $A_{3}=\left( \begin{matrix} 0 & 0 & 0 \\ 0 & 0 & 0 \end{matrix} \right)$, then we obtain

$\det(A_{1}-\lambda I)\det(A_{4}-\lambda I)=0$.

Again this allows us to apply the Routh-Hurwitz stability conditions separately to the matrices $A_{1}$ and $A_{4}$.

According to the Routh-Hurwitz stability conditions we obtain from matrix $A_{1}$

**Condition 1:**

trace$\left( A_{1} \right)<0$,

$-{(\beta}_{r}I_{r}^{^}+\mu+\gamma)-(\alpha_{r}+\mu)-\left( \omega_{r}+\phi_{r}+\tau_{r}+\mu\right)<0$,

**Condition 2:**

$\left| \begin{matrix} -\left( \alpha_{r}+\mu\right) & \beta_{r}S^{^} \\ \alpha_{r} & -\left( \omega_{r}+\phi_{r}+\tau_{r}+\mu\right) \end{matrix} \right|+\left| \begin{matrix} -\left( \beta_{r}I_{r}^{^}+\mu+\gamma\right) & -\beta_{r}S^{^}+\phi_{r}-\gamma\\ 0 & -\left( \omega_{r}+\phi_{r}+\tau_{r}+\mu\right) \end{matrix} \right|+$

$\left| \begin{matrix} -(\beta_{r}I_{r}^{^}+\mu+\gamma) & -\gamma\\ \beta_{r}I_{r}^{^} & -(\alpha_{r}+\mu) \end{matrix} \right|>0$.

Which gives

$$\left( \left( \alpha_{r}+\mu\right)\left( \omega_{r}+\phi_{r}+\tau_{r}+\mu\right)-\alpha_{r}\beta_{r}S^{^} \right)+\left( \omega_{r}+\phi_{r}+\tau_{r}+\mu\right)\left( \beta_{r}I_{r}^{^}+\mu+\gamma\right)$$

$+\left( \alpha_{r}+\mu\right)\left( \beta_{r}I_{r}^{^}+\mu+\gamma\right)+\beta_{r}I_{r}^{^}\gamma>0$,

Substituting in the analytical solution for $S^{^}$ and the expression for $R_{0s}$ we find that the first bracketed term cancels, which yields

$$\left( \omega_{r}+\phi_{r}+\tau_{r}+\mu\right)\left( \beta_{r}I_{r}^{^}+\mu+\gamma\right)+\left( \alpha_{r}+\mu\right)\left( \beta_{r}I_{r}^{^}+\mu+\gamma\right)+\beta_{r}I_{r}^{^}\gamma>0.$$

**Condition 3:**

$\det\left( A_{1} \right)<0$,

$$\left( -\beta_{r}I_{r}^{^}-\mu-\gamma\right)\left( \left( \alpha_{r}+\mu\right)\left( \omega_{r}+\phi_{r}+\tau_{r}+\mu\right)-\alpha_{r}\beta_{r}S^{^} \right)+\gamma\left( -\left( \omega_{r}+\phi_{r}+\tau_{r}+\mu\right) \right)\beta_{r}I_{r}^{^}$$

$+\left( -\beta_{r}S^{^}+\phi_{r}-\gamma\right) \alpha_{r}\beta_{r}I_{r}^{^}<0$,

Once again we find that the first term cancels and the remaining terms can be rearranged to obtain (note that we have divided through by $\left( \alpha_{r}+\mu\right)\left( \omega_{r}+\phi_{r}+\tau_{r}+\mu\right)$ and substituted in $S^{^}=\frac{N}{R_{0r}}$ and $R_{0r}=\frac{N\alpha_{r}\beta_{r}}{(\alpha_{r}+\mu)\chi_{r}}$):

$\left( \frac{\gamma\beta_{r}}{(\alpha_{r}+\mu)}+\frac{\alpha_{r}\beta_{r}(\beta_{r}S^{^}+\gamma)}{(\alpha_{r}+\mu)\left( \omega_{r}+\phi_{r}+\tau_{r}+\mu\right)}-\frac{\alpha_{r}\beta_{r}\phi_{r}}{(\alpha_{r}+\mu)\left( \omega_{r}+\phi_{r}+\tau_{r}+\mu\right)} \right)I_{r}^{^}>0$.

since$I_{r}^{^}=\frac{\mu}{\beta_{r}} \left( R_{0r}-1 \right)\frac{(\gamma+\mu)}{\sigma}>0$.

Remembering the expression of $\sigma$ (equation (S8)), which is positive, we realize that this condition is satisfied if$R_{0r}>1$.

and $\left( \frac{\gamma\beta_{r}}{\left( \alpha_{r}+\mu\right)}+\frac{\alpha_{r}\beta_{r}\left( \beta_{r}S^{^}+\gamma\right)}{\left( \alpha_{r}+\mu\right)\left( \omega_{r}+\phi_{r}+\tau_{r}+\mu\right)}-\frac{\alpha_{r}\beta_{r}\phi_{r}}{\left( \alpha_{r}+\mu\right)\left( \omega_{r}+\phi_{r}+\tau_{r}+\mu\right)} \right)>0$,

which gives,

$\frac{\gamma\beta_{r}}{(\alpha_{r}+\mu)}+\frac{R_{0r}\gamma}{N}+\frac{{\mu R}_{0r}\phi_{r}}{N}+\frac{R_{0r}(\alpha_{r}+\mu)(\omega_{r}+\tau_{r}+\mu)}{N}>0$.

Now from matrix $A_{4}$

$\mathrm{trace}\left( A_{4} \right)<0$,

$-\left( \alpha_{s}+\mu\right)-\left( \omega_{s}+\phi_{s}+\tau_{s}+\mu\right)<0$,

$\left( \alpha_{s}+\mu\right)+\left( \omega_{s}+\phi_{s}+\tau_{s}+\mu\right)>0$.

and

$\det\left( A_{4} \right)>0$,

$\left( \alpha_{s}+\mu\right)\left( \omega_{s}+\phi_{s}+\tau_{s}+\mu\right)-\alpha_{s}\beta_{s}S^{^}>0$,

Which gives

$\frac{\alpha_{s}\beta_{s}N}{\left( \alpha_{s}+\mu\right)\left( \omega_{s}+\phi_{s}+\tau_{s}+\mu\right) R_{0r}}<1$,

which becomes

$R_{0r}>R_{0s}$.

Hence, the Routh-Hurwitz conditions are satisfied when $R_{0r}>{max[1,R}_{0s}]$. Therefore, the mono-existent endemic equilibrium $E^{^}$ is locally asymptotically stable if$R_{0r}>{max[1,R}_{0s}]$, which means that DS TB dies out but DR TB persists in the population.

Figure S1 depicts model trajectories in the $I_{s}$ vs $I_{r}$ plane with different initial conditions using baseline parameter values (see Table 1) for which the disease-free equilibrium is asymptotically stable. In this case both DS and DR TB ($I_{s}$and$I_{r}$) die out, this is because the basic reproduction numbers were less than one (${max[R}_{0s}, R_{0r}]<1$). Figure S2 depicts model trajectories in the $I_{s}$ vs $I_{r}$ plane with different initial conditions using baseline parameter values (see Table 1) for which the mono-existent endemic equilibrium is asymptotically stable. In this system the DS TB strain ($I_{s}$) dies out but DR TB strain ($I_{r}$) persists in the population ($R_{0r}>max[R_{0s},1]$).

Figure S3 depicts model trajectories in the $I_{s}$ vs $I_{r}$ plane with different initial conditions using baseline parameter values (see Table 1) for which the co-existent endemic equilibrium is asymptotically stable ($R_{0s}>max[R_{0r},1]$). In such a case both DS and DR TB ($I_{s}$and$I_{r}$) persist; this is due to the basic reproduction number $R_{0s}$ of DS TB is greater than one and there was an amplification (acquired drug resistance) pathway from DS TB to DR TB.


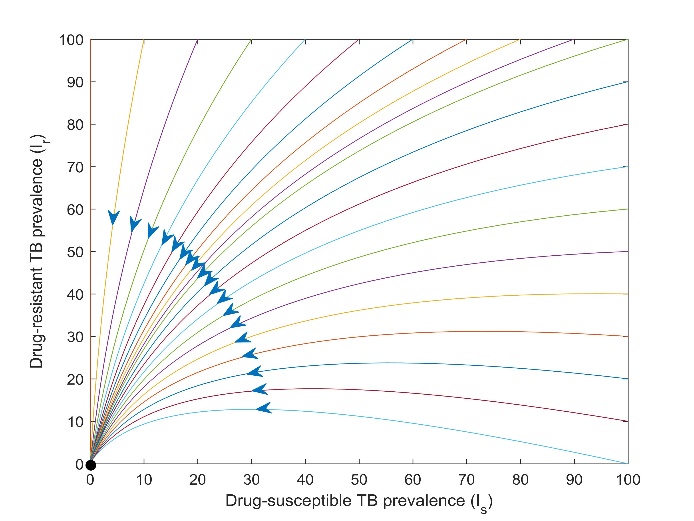


**Figure S1.** Disease-free equilibrium: ${max [R}_{0s},R_{0r}]<1$. The disease-free equilibrium is asymptotically stable, which means that the disease naturally dies out. Here we consider $R_{0s}=0.4$ and $R_{0r}=0.3$.


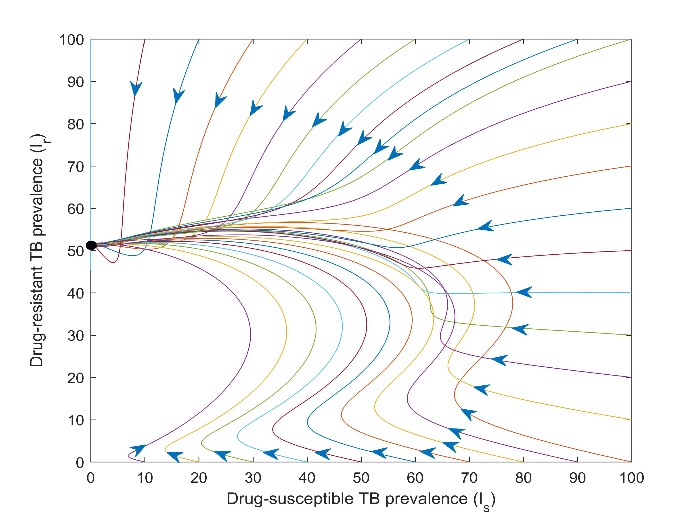


**Figure S2.** Mono-existent equilibrium: $R_{0r}>max[R_{0s},1]$. In this case DS TB dies out but the DR TB persist in the population. Here we consider $R_{0s}=0.4$ and$R_{0r}=3$.


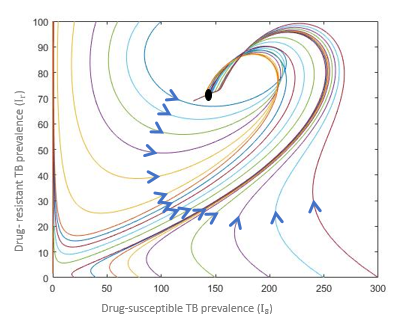


**Figure S3.** Co-existent equilibrium: $R_{0s}>max[R_{0r},1]$. In this both DS TB and DR TB persist in the population. Here we consider $R_{0s}=5$ and$R_{0r}=3$.

**Sensitivity analysis**

Figure S4 displays the correlation between total TB prevalence $(I_{s}+I_{r})$ and corresponding parameters $\beta_{s}, {\alpha_{s},\omega}_{s}, \phi_{s},\tau_{s}, \beta_{r}, {\alpha_{r},\omega}_{r}, \phi_{r}, \tau_{r}, \rho$ and $\gamma$, when $R_{0s}>max[R_{0r}, 1]$. From Figure S4 it is easy to see that total TB prevalence has a positive association with $\beta_{s}, \alpha_{s}, \beta_{r}, \alpha_{r} and \gamma$, suggesting that positive changes in these parameters will increase the total TB prevalence. Further, parameters $\omega_{s}, \phi_{s},\tau_{s}, \omega_{r}$ $, \phi_{r}, \tau_{r}$and $\rho$ have a negative association with total TB prevalence, which refers increasing these parameters values will subsequently decline the total TB prevalence.

Figure S5 displays the association between the DR TB prevalence and corresponding model parameters $\beta_{s}, {\alpha_{s},\omega}_{s}, \phi_{s},\tau_{s}, \beta_{r}, {\alpha_{r},\omega}_{r}, \phi_{r}, \tau_{r}, \rho$ and $\gamma$ when $R_{0r}>R_{0s}$ and $R_{0r}>1$, i.e. at the mono-existent endemic equilibrium. Parameters$\beta_{s}$, $\alpha_{s}, \beta_{r}, \alpha_{r}$, $\rho$ and $\gamma$ have positive PRCC values and parameters $\omega_{s}, \phi_{s}, \tau_{s} \omega_{r}$, $\phi_{r}$ and $\tau_{r}$ have negative PRCC values. Although the parameters values $\omega_{s}, \phi_{s}$ and $\tau_{s}$ are negative, they are negligible because in the mono-existent equilibrium, DS TB dies out and the parameters have insignificant impact on DR TB prevalence.


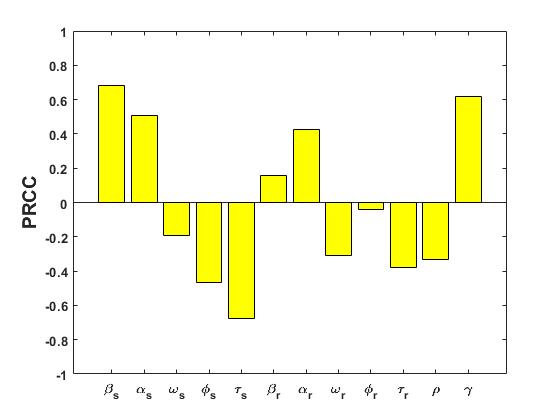


**Figure S4.** PRCC values describing the association between model output $I_{s}+I_{r}$ and the model parameters$\beta_{s}$, $\alpha_{s}$ $\omega_{s}$ , $\phi_{s}, \tau_{s}, \beta_{r}, {\alpha_{r}, \omega}_{r},$ $\phi_{r}, \tau_{r} and \rho$, when $R_{0s}>max[R_{0r}, 1]$.


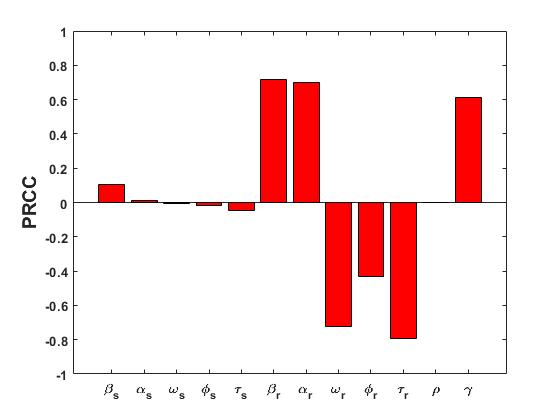


**Figure S5.** PRCC values depicting the sensitivities of the model output $I_{r}$ and the estimated parameters$\beta_{s}$, $\alpha_{s}$ $\omega_{s}$ , $\phi_{s}, \tau_{s}, \beta_{r}, {\alpha_{r}, \omega}_{r},$ $\phi_{r}, \tau_{r} and \rho$, when $R_{0r}>R_{0s}\mathrm{and}R_{0r}>1$.
